# Application of Two-Dimensional Materials towards CMOS-Integrated Gas Sensors †

**DOI:** 10.3390/nano12203651

**Published:** 2022-10-18

**Authors:** Lado Filipovic, Siegfried Selberherr

**Affiliations:** Institute for Microelectronics, TU Wien, Gußhuasstraße 27-29/E360, 1040 Vienna, Austria

**Keywords:** 2D materials, gas sensing, CMOS integration, graphene oxide, transition metal dichalcogenides (TMDs), molybdenum disulfide, phosphorene, MXenes, VOCs, nitrogen dioxide

## Abstract

During the last few decades, the microelectronics industry has actively been investigating the potential for the functional integration of semiconductor-based devices beyond digital logic and memory, which includes RF and analog circuits, biochips, and sensors, on the same chip. In the case of gas sensor integration, it is necessary that future devices can be manufactured using a fabrication technology which is also compatible with the processes applied to digital logic transistors. This will likely involve adopting the mature complementary metal oxide semiconductor (CMOS) fabrication technique or a technique which is compatible with CMOS due to the inherent low costs, scalability, and potential for mass production that this technology provides. While chemiresistive semiconductor metal oxide (SMO) gas sensors have been the principal semiconductor-based gas sensor technology investigated in the past, resulting in their eventual commercialization, they need high-temperature operation to provide sufficient energies for the surface chemical reactions essential for the molecular detection of gases in the ambient. Therefore, the integration of a microheater in a MEMS structure is a requirement, which can be quite complex. This is, therefore, undesirable and room temperature, or at least near-room temperature, solutions are readily being investigated and sought after. Room-temperature SMO operation has been achieved using UV illumination, but this further complicates CMOS integration. Recent studies suggest that two-dimensional (2D) materials may offer a solution to this problem since they have a high likelihood for integration with sophisticated CMOS fabrication while also providing a high sensitivity towards a plethora of gases of interest, even at room temperature. This review discusses many types of promising 2D materials which show high potential for integration as channel materials for digital logic field effect transistors (FETs) as well as chemiresistive and FET-based sensing films, due to the presence of a sufficiently wide band gap. This excludes graphene from this review, while recent achievements in gas sensing with graphene oxide, reduced graphene oxide, transition metal dichalcogenides (TMDs), phosphorene, and MXenes are examined.

## 1. Introduction

Our perception of the environment around us is, to a large extent, shaped by the ambient air and the broad set of gas molecules which can be found in our vicinity [1]. While our noses, or more precisely the olfactory sensory neurons in our noses, are very efficient in detecting the presence of certain gases due to the perceived smell that they give off, they fail entirely in detecting a precise and specific concentration of the inhaled gas and in the sensing of poisonous and potentially lethal gas molecules which do not have a specific odor, such as carbon monoxide (CO). The five primary polluting agents which are classified by the World Health Organization (WHO) as those with the strongest evidence for public health concern include ground-level ozone (O_3_), particulate matter (PM_2.5_ and PM_10_), carbon monoxide (CO), sulfur dioxide (SO_2_), and nitrogen dioxide (NO_2_) [2,3]. Nevertheless, even the most hazardous pollutants are harmful only when their concentration exceeds a specified limit, usually in terms of parts per million (ppm), parts per billion (ppb), or mg/m^3^ in volume. Therefore, many environmental organizations and governments have set recommendations, regulations, and restrictions in place on the permissible quantity of these relevant pollutants in the air, with the ultimate goal to reduce their risk to the health of humans, animals, and the broader environment [4]. The different standards used by the WHO, the European Commission (EC), the United States (US) Environmental Protection Agency (EPA), and the Chinese Ministry of Environmental Protection (MEP) are summarized in Table 1 [4,5].

**Figure 1:**
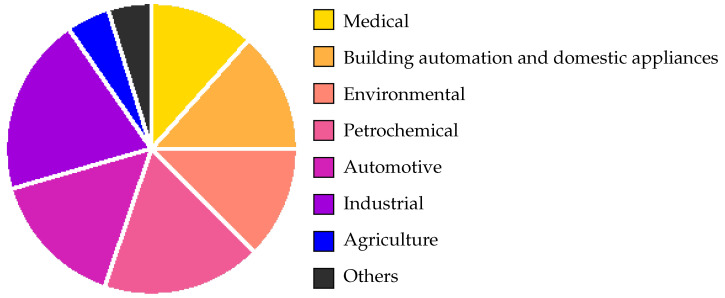
Industries of relevance for the gas sensor market and their global financial shares by end-use in 2021. (source: https://www.grandviewresearch.com, accessed on 26 September 2022).

For a wide variety of applications and industries, it is crucial to be able to identify these and other poisonous and harmful gases in the atmosphere. Among the most important ones include environmental monitoring [6,7,8,9], health and safety [10,11], automotive and transport [12,13], and chemical warfare detection [14,15]. Therefore, it is not surprising that the global gas sensor market is quite sizeable. In Figure 1, which displays the global gas sensor market share by end-use, we observe that the end-use applications are rather evenly distributed throughout a number of applications and that no specific industry is heavily dominating sensor development [16]. It is quite clear that the industry, in a broad sense, depends on sensors to detect a large set of polluting gases at varying concentrations. People have been interested in gas detection for many years, and before the development of gas sensors, several gases were detected by animals for specific applications: For example, when a canary is exposed to carbon monoxide, carbon dioxide, or methane, it stops singing, which made it a common tool for gas detection in mines. The current gas sensor market is valued between one and two billion euro and is expected to increase with an annual growth rate of about 5–10% during the next 5 to 10 years [16,17]. In industrial applications, there will be an increasing need for gas sensors in the oil and gas industry for process optimization, in the further development of Internet of Things (IoT) and Internet of Everything (IoE), in smart appliances, smart cities, self-driving vehicles, non-invasive disease detection, and many other areas. However, there are still several technical issues to overcome, in addition to the high costs associated with commercial gas sensors today [18].

The predicted gas sensor market expansion comes from the increased need for gas sensor integration with vital communication technologies to enable the aggressive advancement of IoT, IoE, cloud computing, etc. The IoT is a multi-layered technology which connects diverse hardware—smart appliances, smart gadgets, wearables, and mobile consumer devices, all of which are equipped with sensors—together with the Cloud of Things (CoT) [19]. Currently, the most significant hurdle to the widespread integration of gas sensors is their price, so the increased application of these devices will only be enabled by unit cost reduction which is achievable through sensor miniaturization and integration with signal drive and processing circuitry [20]. This most often means the use of solid-state and semiconductor-based gas sensors with integration with a low-cost and mature manufacturing technology, such as complementary metal oxide semiconductor (CMOS) fabrication [9].

With a focus on chemiresistive sensors, based on semiconductor materials which demonstrate the greatest potential for future CMOS integration and miniaturization, we build on the review we presented at the 32nd International Conference on Microelectronics (MIEL) in 2021 [1] and summarize some key aspects of currently available gas sensor technologies. Our analysis results in the conclusion that the most likely materials which have the potential for both sensing applications and digital logic are two-dimensional (2D) materials, and we expect this integration in the relatively near future [21,22]. First, we introduce different gas sensing technologies, their integration with CMOS processes, and the current research into room-temperature gas sensing. This includes a summary of the current chemiresistive gas sensing workhorse, mainly the semiconductor metal oxide (SMO) sensor, and the principal concerns behind this technology. Subsequently, we summarize the main types of gas sensors which are based on semiconductor materials and which have the potential for CMOS integration: Chemiresistive and field-effect transistor (FET) sensors. After this, we dive into the current research on the synthesis of 2D materials, whereby we examine its potential for the integration of these methods and processes in a CMOS foundry. Finally, we summarize several advancements in the application of various 2D materials for gas sensing.

## 2. State-of-the-Art in Gas Sensing Technologies

The demands for today’s IoT sensors are very ambitions and call for a number of desirable characteristics, such as [20]:

1. Cost-efficient fabrication and operation;
2. Reduced power dissipation;
3. Improved repeatability and long-term reliability;
4. Capability of real-time communication;
5. Heightened data security.

In general, it is accepted that the integration of a sensor technology with CMOS foundry and digital logic is a step in the right direction towards IoT devices. Specifically, cost-efficient fabrication and operation, power dissipation reduction, and repeatability and long-term reliability can be directly achieved through integration with CMOS fabrication. Real-time communication and heightened data security could be helped along by the integration of the sensing response with digital, analog, and radio-frequency (RF) components, as it would allow signals to be processed directly on the chip, making it fast and allowing for encryption to take place before the signal is transmitted.

In this section, we look at currently available gas sensing principles, the state-of-the-art CMOS integration of gas sensors, the application of SMOs and other semiconducting materials for gas sensing, and the current technologies with the potential for room-temperature sensing. Two-dimensional materials are kept out of this discussion, as they are still not quite a mature technology and we will analyze these in more detail in later sections.

### 2.1. Gas Sensing Principles

A large set of materials for gas sensing and gas sensor designs are under investigation and find themselves at vastly different maturity levels of realizable development. Several advanced gas sensing technologies which have already been commercialized by industry include electrochemical (EC) sensors, catalytic pellistors (CP), thermal pellistors (TP), piezo-electric (PE) sensors, photo-ionization (PI) devices, optical infrared (IR) adsorption sensors, and SMO chemiresistors [9,23,24,25]. These technologies are typically divided into two categories: One whose detection mechanism is based on changing a material’s electrical behavior after adsorption (e.g., conductivity, field effect) and a second whose detection depends on an induced change in another property (e.g., thermal, optical) [26]. Several important properties of these types of sensors, including advantages and disadvantages, are summarized in Table 2.

**Figure 2:**
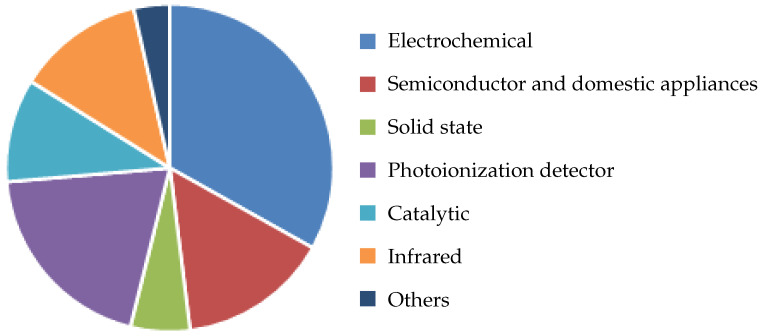
United States gas sensors market share by technology in 2019. (source: https://www.gminsights.com, accessed on 26 September 2022).

The most relevant conclusion we can make from Table 2 regarding the SMO sensor is that this semiconductor-based chemiresistive technology provides an option with the lowest cost, footprint, and power dissipation, mainly as a consequence of its successful integration with CMOS fabrication techniques. As already alluded to earlier, these characteristics are necessary in order to enable sensing solutions for portable technologies as well as IoE and IoT integration, while integration with CMOS further ensures a means for very high and reliable reproducibility [27]. It is crucial for the volume manufacturing of commercial devices that there be minimal inter-device variances and that there is high confidence in the capability to produce a device with predictable attributes and highly manageable tolerances. We also observe a low power dissipation attributed to the catalytic pellistor which is also inexpensive to manufacture and has a comparatively small physical footprint. However, compared to the SMO sensor, this device has far worse selectivity, lesser sensitivity, and a slower reaction time. Piezoelectric, photo-ionization, and infrared sensors all offer excellent sensitivity, but they all have the drawback of high power consumption, which prevents portability and IoT integration. There has been some progress in reducing the price of IR sensors in the wavelength range for CO and CO_2_ detection, but this is still nowhere near the requirement of mass-market integration, which is discussed further in Section 2.2. As shown in Figure 2, the electrochemical sensor has the biggest market share in the US and appears to be a reasonable all-around option. However, it is difficult to integrate this type of sensor into portable technologies. It only has a six to twelve month shelf life, and both high temperatures and low humidity cause the drying out of the electrolyte [28,29]. Other potential solutions, based on semiconductor materials and CMOS-friendly fabrication techniques are, therefore, readily investigated and sought after.

In comparison to existing alternatives, the SMO sensor appears to provide the most benefits towards potential IoT applications. Previous research has shown that it has several advantages leading to its commercialization [30,31,32,33,34,35,36,37], particularly in terms of response time, sensitivity, and possibilities for portability and down-scaling. The high sensitivity of SMO sensors is, however, only made possible by applying very high operating temperatures, which may compromise their reliability and durability. With the goal of CMOS integration for a chemiresistive gas sensing solution, the SMO sensor has emerged as the industry standard. A semiconductor-based approach, which operates at ambient temperature and can be designed with established CMOS fabrication techniques is, therefore, highly desired. Several sensors and prospective sensing materials, including 2D semiconductors such as graphene and transition metal dichalcogenides (TMDs), are being investigated in this direction [38]. The fabrication of these films, and devices based on these films, is not trivial; however, investigations in the past few years have shown some promise in fabricating films of high quality for sensing and have made significant progress [39,40].

### 2.2. CMOS-Gas Sensor Integration

Further growth can only be generated for semiconductor-based gas sensors if they can be fabricated on a CMOS chip and combined with the associated circuitry using standard microelectronic processes at reasonable costs. The reasonable cost is estimated to be in the range of USD 1–USD 2 to access new high-volume and portable markets, while current sensors range in price from about USD 50 for silicon-based sensors to well over USD 70 for optical solutions [41]. In order to ensure proper integration with CMOS technology, research into gas sensors is most often restricted to chemiresistive or FET solutions with an electronic amplification and read-out circuit. The combined integration of the fabrication of these devices with mature CMOS technology with an on-chip signal conversion and amplification is the ultimate goal for low-power and portable technologies. As noted in the previous section, the main type of sensors which has been successfully integrated with CMOS fabrication for a single-die solution and for low-cost fabrication is the resistive SMO sensor. The main challenges are to deposit the required films without infringing on the restrictions imposed by a CMOS foundry and to isolate the sensing area, when heated, to the rest of the electronics [41].

**Figure 3:**
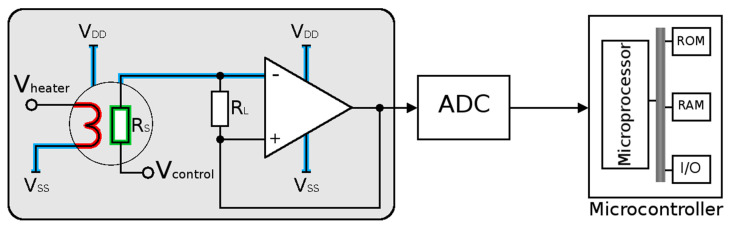
Typical schematic of a single SMO sensor with interface blocks. The sensor requires a heating element, a voltage follower, and an analog-to-digital converter (ADC). In order to analyze the obtained data, it is passed to a microcontroller, which is enabled with a read-only memory (ROM), random access memory (RAM), and input/output (I/O) interfaces.

A typical resistive SMO sensor circuit is shown in Figure 3. The microheater (red), sensing element (green), critical interconnects (blue), and analog and digital circuitry are all on a single system-on-chip (SoC) implementation [42,43]. The complexity of such an integration is immediately evident. The critical component is the integration of the microheater, since this needs to provide a high temperature to the sensing film while not disturbing the temperature of the nearby digital, analog, or RF components. An additional challenge is to reduce the sensor size while making sure that the microheater provides a stable and uniform temperature over the entire sensor area without the threat of total mechanical failure [44].

**Figure 4:**
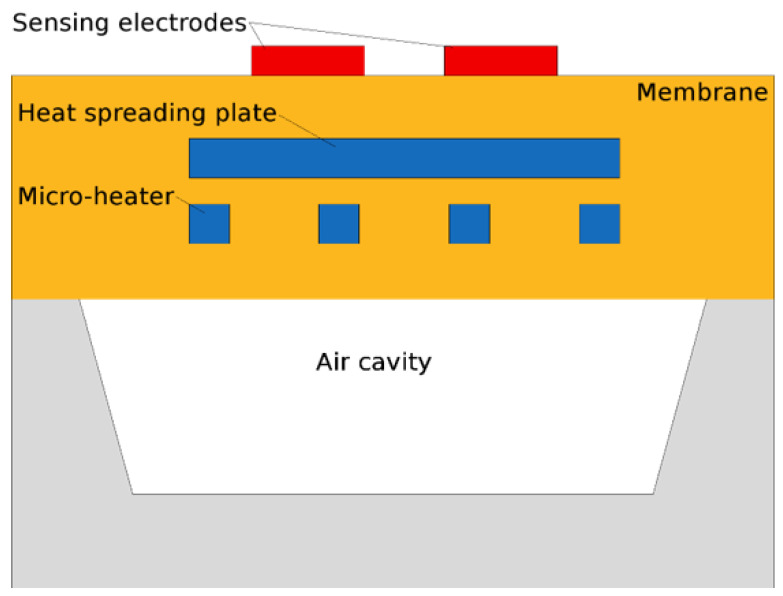
Cross-section schematic of the layers composing the membrane of the hotplate.

**Figure 5:**
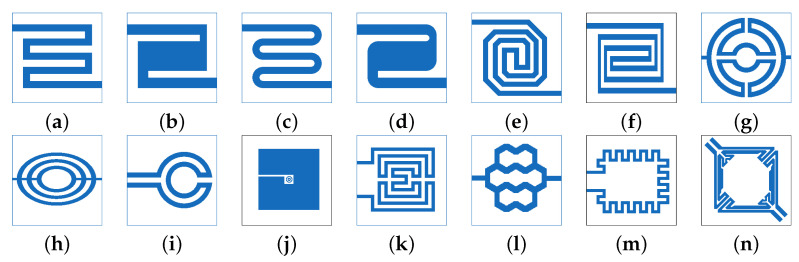
Microheater geometries characterized and modeled over the last decades. The shapes depicted are: (**a**) Meander, (**b**) S-meander, (**c**) Curved, (**d**) S-curved, (**e**) Double spiral, (**f**) Square double spiral, (**g**) Drive wheel, (**h**) Elliptical, (**i**) Circular, (**j**) Plane plate, (**k**) Fin shape, (**l**) Honeycomb, (**m**) Loop shape, (**n**) Irregular.

To integrate the microheater on a silicon wafer, a thin, thermally isolated membrane, most often composed of stacked layers of SiO_2_ and SiN, is desired to reduce power loss. The microheater element is then incorporated inside this membrane, while the sensing film and electrodes are placed on top, cf. Figure 4. The shape of the microheater can vary drastically and is a design decision based on power and performance; a few options which have been tested experimentally and theoretically are given in Figure 5. The choice of microheater material is also critical, since early attempts to use CMOS-friendly materials such as polysilicon [45] and aluminum [46] were both met with reliability issues. Polysilicon suffers from poor long-term stability at high temperatures because of electromigration, grain boundary motion, and high crack propagation. Aluminum likewise suffers from electromigration at high temperatures, and its relatively low melting point softens the material at elevated temperatures. Platinum heaters are highly stable at 400 °C, but the material is not native to a CMOS foundry, meaning that it has to be deposited with a post-CMOS step. It should be noted that most authors who claim to provide a “CMOS compatible” fabrication typically require non-standard materials or processes, meaning that, realistically, a modified CMOS flow is proposed [47].

Worldwide, there are very few groups who have attempted to apply combined commercial CMOS and microelectromechanical system (MEMS) foundry processes to fabricate in-chip sensor and circuit solutions. A few of these are the group of Prof. Udrea from Cambridge University [48], Prof. Gardner from Warwick University [49], and Prof. Baltes from ETH Zurich [50]. The main idea stemming from Cambridge University is to use a silicon-on-insulator (SOI) CMOS wafer with a tungsten heater [51] and aluminum metalization. The membranes were formed using backside deep reactive ion etching from a MEMS foundry, ensuring that a 300 °C temperature can be reached at 6 mW of power. The other research groups have achieved similar successes using a non-SOI wafer and a silicon-based microheater, which either uses silicon plugs or a metal-oxide semiconductor field-effect transistor (MOSFET) heater [52,53]. The last stage in the design of these sensors is the deposition of the SMO sensing layer, which is then exposed to the ambient environment, ready to react to the presence of desired gas molecules; this is discussed further in the next section.

### 2.3. Semiconductor Metal Oxide Gas Sensors

**Figure 6:**
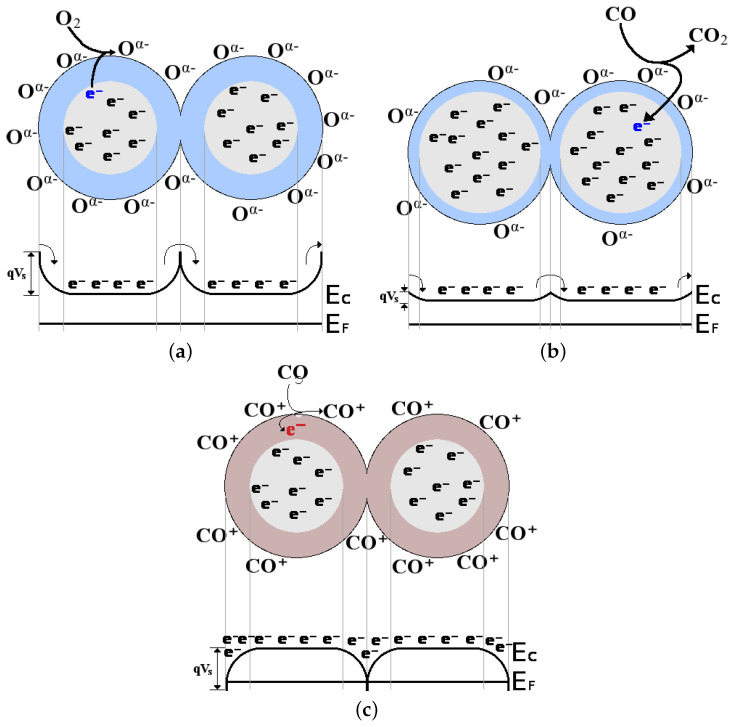
Gas sensing and resulting band bending for a granular SMO film where (**a**) oxygen adsorbs onto the surface, creating a depletion region; (**b**) the adsorbed oxygen reacts with CO, reducing the amount of band bending; and (**c**) CO adsorbs directly on the surface without the presence of oxygen, forming an accumulation region.

While our aim is to present the advancements made in gas sensing with 2D materials beyond graphene, the discussion would be incomplete without an understanding as to why 2D materials are of such high importance and why they are so heavily investigated for sensing applications. The main reason is the limitation of the current standard for chemiresistive gas sensing, which relies on the use of SMO films [38]. There are multiple means of interaction between the exposed surface and grains of an SMO film and a nearby gas molecule:

1. In an inert ambient, e.g., N_2_, the energy bands at the surface are flat and no depletion or accumulation region can build up. The number of charges at the surface is the same as that in the bulk film.
2. When oxygen is introduced in the environment (or from the oxygen in the air) the vacancies on the surface of the SMO film are populated by the adsorption of O^−^ or O_2_^−^. Thereby, the bulk film donates one or two electrons to the adsorbed oxygen, respectively, forming a depletion region near the surface. This depletion region results in band bending, depicted in Figure 6a.
3. When a reducing gas (e.g., CO) enters the ambient environment together with oxygen, it will react with the adsorbed oxygen on the surface, thereby removing it to form CO_2_, cf. Figure 6b. In the presence of O_2_ and CO, the surface will continuously re-oxidize, leading to a reduction in the depletion region, which depends on the concentration of CO molecules in the ambient.
4. It has also been shown that, even without the presence of oxygen, some gas molecules will adsorb at the surface vacancy sites, ultimately reducing the surface, cf. Figure 6c. In this interaction, the CO molecule donates an electron, forming an accumulation region.

To provide sufficient energy to the SMO surface which serves to enable oxidation-reduction reactions with gas molecules, the SMO-based sensor has to operate at high temperatures, i.e., between 200 °C and 550 °C [9]. The high temperature requirement necessitates that the sensing film is integrated with a microelectromechanical system (MEMS) microheater. This microheater, due to the high temperatures it produces, must also be thermally isolated from other devices and components, including RF, analog, and digital circuits [44,54]. The added complexity in the geometry of the sensing device, which requires the use of a suspended membrane to host the microheater and sensing film, is a further concern regarding long-term reliability due to the inherent thermo-mechanical stability problems associated with large temperature variations [55]. The device’s repeated heating and cooling can cause a buildup of thermally induced mechanical strain, which can eventually cause cracking, delamination, and the sensor to completely fail mechanically. In addition, the microheater itself comes with several reliability concerns such as electro- and thermo-migration [56]. Furthermore, due to the type of surface reaction taking place during sensing, the SMO film is highly sensitive to oxygen and humidity in the ambient environment, making it difficult to design a volatile organic compound (VOC) sensor, as these are commonly accompanied by high relative humidity.

Various scientific groups are actively investigating the possibility to fabricate a semi-conductor-based, CMOS-integrated, chemiresistive gas sensor device which is able to operate at low temperatures while simultaneously ensuring a reasonably high specificity and selectivity towards target gases [38,57,58,59]. Graphene, TMDs, and other two-dimensional (2D) materials have already displayed some promise towards gas sensing operation at room temperature (RT). This is mainly due to their inherently high surface-to-volume ratios [11]. The high ratio ensures that the surface which is exposed to a target gas, and which in-turn affects the conductive behavior of the bulk film, is sufficiently large to provide low sensing limits. This will be discussed in more depth in Section 4.2.

### 2.4. Other Semiconductor Materials for Gas Sensing

**Figure 7:**
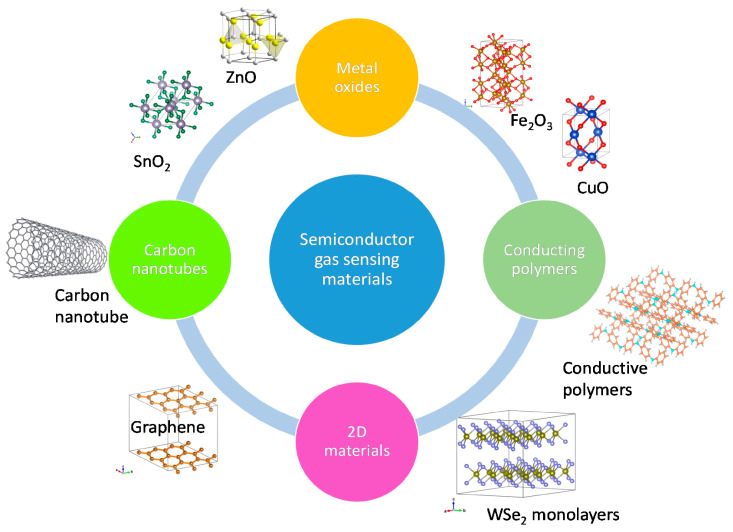
Visual representation of semiconducting films that are being investigated for their potential in gas sensing devices. (Reprinted with permission from Nikolić et al. [20], CC-BY 4.0).

For many years, there has been a significant amount of research dedicated to various semiconducting films which have the potential of being employed in gas sensing applications. Because of the limitations in selectivity and the need for high-temperature operation of SMO films, as discussed in the previous section, research has increasingly been moving towards alternative materials, such as SMO nanostructures [24,60,61], conducting polymers [62,63], carbon nanotubes [64,65,66], and, most recently, 2D materials [7,67,68,69]. A general review of the various semiconducting films studied due to their high potential for gas sensor applications is given in Figure 7 [20]. With the exception of SMO films, the materials which are shown are largely in the early research phase, and the commercialization of these films has not yet taken place.

The potential industrial applications of conducting polymers are limited by their difficult and time-consuming fabrication processes [70]. Furthermore, as a consequence of oxidation, the long-term durability of gas sensor devices which are based on conducting polymers is much lower than that of gas sensors based on SMO films [20]. Cylinder-shaped carbon nanotubes (CNTs) are produced by the wrapping of pristine graphene sheets along the axial direction [71]. These layers provide a high surface-to-volume ratio, strong chemical and mechanical stability, and appropriate electronic characteristics [72]. However, because it is challenging to manufacture continuous nanotubes free of defects and because their production is incompatible with CMOS technology, their potential for use in the future may be limited by an expensive synthesis [73,74]. As a result of the propensity of CNTs to energetically bond with oxygen and water molecules, thereby affecting its sensing response, an additional issue is a lack of selectivity and specificity.

There is a plethora of further studies on exotic applications of CNTs and structures with enhanced properties for gas sensing, portability, or low-power operation [75]. Similar to the CNT, graphene-based yarn architectures have been shown to be a potential solution for self-powered photoelectrochemical sensing of methane [76]. CNTs have also been interwoven in three-dimensional (3D)-printed fabrics to spearhead a new generation of portable electronics [77]. While there is promise in the future use of these materials in a wide range of applications, their fabrication is far from what is required for CMOS integration. A more thorough look at CNT-based devices and potential for their synthesis and future applications is provided by Rathinavel et al. in [78].

In order to facilitate the integration of the sensing materials with analog and digital electronics, and to make the synthesis of the semiconducting sensor films compatible with mass-produced low-cost digital transistors, which are the main driver of the microelectronics industry, the chosen solution must be compatible with digital CMOS transistor fabrication. In light of this, 2D materials seem to have the most promising chances for their potential application in a variety of imagined devices and for commercialization in the relatively near future. Another benefit of 2D materials is their high sensitivity, even at room temperature. Therefore, before we dive into the topic of 2D based sensors, the next section will look at currently investigated solutions for low-temperature and room-temperature gas sensing.

### 2.5. Room-Temperature Gas Sensing Solutions

As mentioned previously in Section 2.3 one of the main drawbacks of the SMO sensor is that it must be heated up to several hundred degrees Celsius in order to provide sufficient energy for the required surface sensing reactions. Therefore, researchers are regularly investigating potential means to reduce the operating temperature down to room temperature. Two-dimensional semiconducting materials appear to provide high potential for this, which is why we discuss these in this review. However, before we delve into the topic of 2D-material-based gas sensors, in this section we look at potential alternatives for room temperature operation. These methods are related to sensing based on an optical response or by introducing an additional light activation to provide the necessary surface energy instead of only elevating the temperature. Note that any involvement of light in the sensing mechanism makes CMOS integration quite challenging, as it requires a directed light source at a specific wavelength or a narrow band of wavelengths. This likely requires different materials with a precise arrangement and complex packaging, which is unlikely to be feasible for on-chip integration.

#### 2.5.1. Nondispersive Infrared Gas Sensors

**Figure 8:**
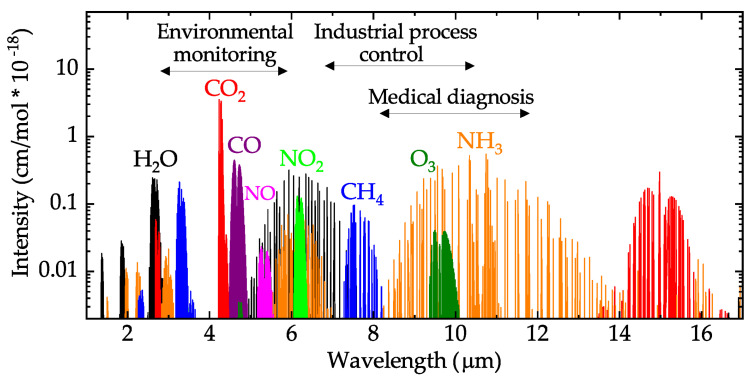
Adsorption spectra in the mid-infrared range of several molecules, including the pollutants from Table 1, and their intensities. (Reprinted with permission from Popa and Udrea [81], CC-BY 4.0).

While this review mainly concentrates on devices where a chemical surface reaction induces a change in the electrical properties of a film, meaning a chemiresistive or FET-based sensor, we would be remiss if we did not mention the recent advances made in optical gas sensing. The most significant and important advantage of nondispersive infrared (NDIR) sensors over all other options is their selectivity, meaning that the sensor provides distinguishable absorption properties of the ambient gas species [79]. The NDIR gas sensor is the most promising optical sensing technique since most toxic and polluting gases exhibit strong absorption in the mid-infrared regime, with wavelengths in the range of 2.5 μm to 14 μm, as shown in Figure 8. While there is much to be excited about with NDIR sensors, there are still several shortcomings with this technology, including their bulky nature due to the need for a lens system, the interference between the overlapping spectra of two gases, and the experimental limit of detection [80].

The basic principle of operation of the NDIR gas sensor is to provide a mid-IR signal which can then interact with ambient gas molecules and, subsequently, to compare the signal strength before and after this interaction. This follows the Beer-Lambert law, given by [82]

(1) $$I\left(\lambda \right)=I_{o}{\left(\lambda \right)e}^{-\alpha \left(\lambda \right)cl},$$

where $I\left(\lambda \right)$ and $I_{o}\left(\lambda \right)$ are intensities of the detected and emitted radiation, respectively, at wavelength λ, while $\alpha \left(\lambda \right)$ is the absorption coefficient of the gas with *c* its concentration and *l* the path length. As could be inferred from the discussion thus far, this device requires several components, mainly the IR emitter, IR detector, and the gas cell, where the IR signal can interfere with the gas molecules. Fabricating the entire device with CMOS technology and integrating it with a digital CMOS logic circuit is not possible. However, recent advances have made it possible to fabricate specific components using CMOS fabrication technology, for specific wavelength ranges.

The most critical component for NDIR gas sensors is the emitter, as it must be able to provide the specific range of wavelengths dependably. These typically come in three flavors: Microbulb lamps, MEMS microheaters, and light-emitting diodes (LEDs) [83]. The microbulb lamp is essentially a thermal emitter and the wavelength which is produced can only be controlled by the produced temperature of the emitter element. One problem is that for applications in the fingerprint region of wavelengths (e.g., 6 μm to 15 μm), a thermal emitter would need to be operated at room temperature, meaning that the emissivity would be very low. This means that novel and complex materials need to be involved and engineered for improved performance. One such option was recently developed for ethanol (9.5 μm) and CO_2_ (4.3 μm) detection using a single wire Kanthal (FeCrAl) alloy filament [84,85].

It is quite evident that the thermal lamps are not CMOS compatible, which is why the use of MEMS microheaters to generate the IR signal has grown in popularity in recent years. These emitters use silicon or SOI wafers to define membrane-based suspended microheater structures, not unlike those required for the SMO sensor described in Section 2.2. MEMS IR emitters are manufactured using CMOS-compatible, high-volume semiconductor technologies, which are cost-effective for mass production [85,86]. The microheater material is most commonly platinum, which is not usually found in CMOS technology, but recent studies have shown the potential of using tungsten instead, which ensures smoother CMOS integration [81]. The MEMS-based microheater IR emitter has shown to be able to produce signals at a broad range of wavelengths, including 4.26 μm for CO_2_ detection [87]. The authors in [88] presented a narrowband NDIR gas sensor for acetone and ammonia detection at wavelengths between 8.26 μm and 10.6 μm. Nevertheless, this type of emitter has similar issues as the SMO sensor regarding thermal stressing and the MEMS membrane’s mechanical stability. Furthermore, the rest of the required elements of the complete gas sensor, such as the IR detector and gas chamber, would still need to be added, and this cannot be fully integrated on a single CMOS chip.

The most recent low-power and low-cost push toward design solutions for NDIR emitters has been the LED design. Advances in semiconductor fabrication and introduction of novel materials have made it possible to develop light-emitting diodes in the IR spectrum [83]. These types of emitters come with the benefit of improved signal-to-noise ratio, improved accuracy, less stabilization issues, and potential for CMOS-friendly fabrication [79]. CO_2_ sensors using narrow-bandgap semiconducting III-V films have already been commercialized [89,90]. A methane sensor was also developed using an LED-based IR emitter, working at a wavelength of 1.65 μm in order to avoid cross-sensitivities to other gases at higher wavelengths. Even with all the benefits that LED IR emitters provide, the fabrication challenge and potential for integration with CMOS digital logic on the same chip is quite limited, compared to the chemiresistive or FET-based solutions. The structure of the LED emitters is quite different from conventional photodiodes, which are based on electron–hole recombination. The requirement of a narrow bandgap material increases Auger recombination, reducing the efficiency at the high carrier injection levels required for occupancy inversion [83]. To overcome this, the light from these LEDs is achieved by recombining electrons and holes in heterostructures, similar to interband cascade lasers. The most recent progress has seen the fabrication of a structure with 16 stages, where electron–hole recombination can take place, in the form of a superlattice AlAsSb/InAs/GaInSb/InAs emission layer and an n/p GaSb/AlInAsSb tunnel junction [91]. The authors therein presented a working LED emission structure with a power output of 6.8 mW at a wavelength of 4.2 μm, relevant for CO_2_ sensing.

#### 2.5.2. Light-Activated Gas Sensors

**Figure 9:**
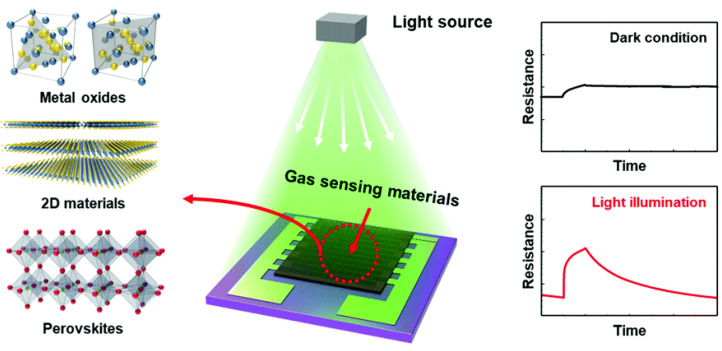
Schematic of a light-activated gas sensor to achieve low power consumption and fast photodesorption after a sensing cycle. (Reprinted with permission from Suh et al. [105], CC-BY 3.0).

As we already mentioned in Section 2.3, the chemiresistive SMO gas sensor needs to be heated in order to provide sufficient energy to initiate the surface reactions during detection. This means that a heater is required, as discussed in Section 2.2. However, other studies propose to either replace or accompany the heater with photostimulation of the sensing surface [92,93], as shown in Figure 9. This was shown to reduce the response time by stimulating the SMO’s recovery from gas adsorption back to the baseline. Most of these studies used ultraviolet (UV) light together with an SMO layer at low temperatures, down to room temperature [94]. For example, Al/Al_2_O_3_ and Al/TiO_2_/Al_2_O_3_ sensors on p-type silicon substrate were demonstrated to operate at room temperature under UV illumination for NO_2_ detection with a near-linear response [95].

The improved low-temperature performance of light-enhanced chemiresistive SMO sensors was attributed to the fact that photogenerated electrons and holes are able to interact with the adsorbed O_2_ and adsorbed gas molecules. For oxidizing gases, such as NO_2_, which could react directly with adsorption sites on the SMO surface, the presence of surface oxygen (i.e., O_2_^−^) under dark conditions meant that less surface area was available for NO_2_ adsorption, meaning O_2_ and NO_2_ were competing for space. Through light irradiation, two processes are initiated, which promote NO_2_ detection: First, photoconductivity is introduced by the formation of electron–hole pairs and the increase in free charge carriers. Second, the light irradiation increases the adsorption/desorption rates, leading to an increased speed and a faster facilitation of new surface adsorption sites through desorption, thereby increasing sensitivity [96,97,98].

Over the years, many publications have demonstrated the suitability of low-power UV LEDs to activate the surface sensing mechanism of many SMO films at low or room temperatures, such as SnO_2_ [99], In_2_O_3_ [100], ZnO [101], WO_3_ [102], and TiO_2_ [103]. While many authors noted a clear increase in the sensitivity response, the SMO sensors nevertheless—whether UV-enhanced or not—still suffered from poor selectivity when a single metal oxide material was used. Therefore, research turned more towards the impact of UV-enhanced sensing with SMO heterojunctions. Investigations are ongoing to study the impact of UV-activated gas sensors with chemical modification of the oxide matrix using catalitically active nanoparticles of noble metals, such as gold, platinum, palladium, or with clusters of PdO, transition metals, or carbon materials [104].

Due to the complexity and power requirements of UV LED integration, further developments concentrated on using visible light for gas sensitivity activation. LEDs which are based on InN/GaN/AlN heterostructures are most commonly applied as a source of UV radiation; however, these have a low efficiency and low quantum yield compared to LEDs which emit visible light, such as those based on InGaAs/SiC semiconductors [104]. In fact, activation using blue light (wavelength range 400 nm–500 nm) and red light (wavelength range 500 nm–600 nm) were shown to be the most efficient for this application [106]. These can often be fabricated using GaN/SiC semiconductors [107,108].

## 3. Semiconductor-Based Gas Sensor Types

**Figure 10:**
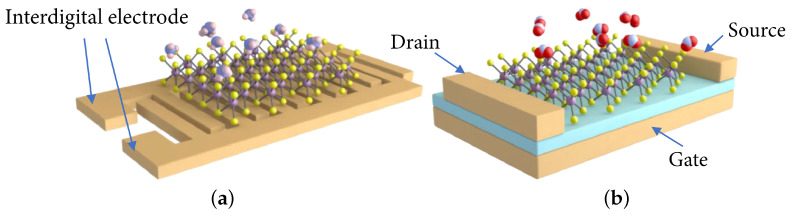
Schematic representation of gas sensors using (**a**) a chemiresistive configuration and (**b**) a back-gated FET configuration. (Reprinted with permission from Cao et al. [109], CC-BY 4.0).

In the previous section, the discussion mainly led to the conclusion that semiconductor solutions to gas sensing are desired, as these provide the easiest integration with modern electronics and the highest potential for efficient fabrication, mainly using established CMOS foundry flows. However, semiconductor-based sensors come in several variations in design and architecture. The most straight-forward type is the chemiresistor which is effectively a variable resistor, whereby the resistance of the semiconductor strip changes as an effect of molecular adsorption on its surface, shown in Figure 10a [109,110,111,112,113,114]. A chemiresistive sensor can also be based on the conduction in a thin nanowire [115,116]. Alternatively, a FET-based gas sensor, sometimes referred to as a thin-film transistor (TFT) gas sensor, is also commonly applied, whereby a back gate is used to control the electrostatics of the channel, effectively allowing (ON-state) or preventing (OFF-state) current flow between the source and drain, shown in Figure 10b [109,114,117,118,119,120]. FET-based sensors can also be designed using a tunnel FET structure or a nanowire channel in order to provide more control over the conduction in the channel. Furthermore, semiconductor films have also been applied in designing inductive and optical gas sensors, as was mentioned in Section 2.5.1; nevertheless, when 2D materials are discussed in terms of gas sensing, it is the chemiresistive and TFT FET devices which are of primary interest [114,121].

### 3.1. Chemiresistors

For chemiresistors, the electrical resistance of the sensor changes as a consequence of the adsorption of gas molecules on its surface. The sensitivity *S* towards a particular gas is, therefore, determined by how much the resistance of the film changes after exposure to this gas, usually starting from a baseline using operating conditions in pure ambient air. However, it should be noted that some studies use inert ambient, e.g., N_2_, as the baseline resistance, which is why it is often difficult to truly compare sensitivities across a broad set of experimental literature studies. From all of the available types of semiconductor-based gas sensors, the chemiresistor is the easiest to fabricate, since it only requires placing the sensing film on top of an insulating substrate and two metal contacts, which are often formed using interdigitated electrodes, cf. Figure 10a. The read-out circuit for the chemiresistive gas sensor is also quite straight-forward since only the current flowing through the resistor needs to be measured, which can be performed by placing a load resistor and reading the voltage across it [122,123]. The ease of chemiresistive gas sensor fabrication brings about the potential for their cost-efficient fabrication, ability of scaling, and eventual CMOS integration.

The key to introducing a new material for chemiresistive gas sensing in a CMOS technology relies on its integration with the back-end-of-line (BEOL) process [124]. The front-end-of-line (FEOL) is primarily reserved for manipulating the silicon wafer in the form of doping and implantation in order to form transistors for memory and digital logic. During BEOL fabrication, the metallization and interconnections between the FEOL devices are created, which is why it is more convenient to introduce new materials, as there is already sufficient insulation to the silicon wafer and metallization is available. Therefore, in principle, it should be relatively easy to introduce a new material, such as a 2D layer within the BEOL steps. However, one major problem here is that most studies which are performed on 2D materials for chemiresistive gas sensing on a silicon wafer rely on a thermally grown SiO_2_ interface to the 2D film. A major problem is that thermally growing SiO_2_ during BEOL is not permissible, since there is a strict thermal budget which must be followed during these steps, and the temperature must not exceed about 400 °C. Therefore, alternatives must be found, such as plasma-enhanced chemical vapor deposition (PECVD)-grown SiO_2_ or SiN insulation on top of which the 2D film is placed [125]. Alternatively, new insulating materials could be introduced in the CMOS flow, but this may further complicate the fabrication, adding more time and cost. The fact that it is not clear which insulator and 2D material combination would make the most feasible integration is an additional concern, since it has become abundantly clear that the choice of insulator plays a very important role in the electrical properties of the 2D film due to the introduction of surface optical phonon scattering, which becomes the dominant scattering effect, limiting the film’s conductivity [126]. It is therefore not clear what impact the rougher PECVD surface will have on the electrical and sensing properties of the chemiresistive film. One option is to introduce a chemical mechanical polishing (CMP) step in the BEOL process to smoothen the insulator surface before introducing the 2D film, something similar to what was proposed by Han et al. in [127]. Nevertheless, a thorough investigation of all materials which contact the 2D film will have to be made before the relatively simple integration of 2D materials for chemiresistive gas sensors are introduced in the BEOL. The synthesis and deposition of the 2D film itself in the CMOS flow also comes with its own specific challenges and is addressed in further detail in Section 4.1.

### 3.2. Field Effect Transistors

The field-effect transistor (FET) is the workhorse of the semiconductor industry and is the cornerstone of digital logic and many memory devices. In addition, the field effect, as implemented in a FET or a MOSFET, has been extensively used in advanced bio-sensing techniques, using biologically sensitive field-effect transistor (BioFET) [128,129] and ion-sensitive field-effect transistor (ISFET) [130,131] designs. These devices provide a means to adjust the electrostatics of the channel layer using an electrode, which is suspended in an electrolyte solution, which is in contact with the gate dielectric. The sensitivity of the BioFET and ISFET structures are governed by how the charge accumulation changes on the gate dielectric and the selectivity is introduced by placing immobilized receptors on top the gate, which will only react to specific bio-molecules. However, generating such a structure, which has a high sensitivity and selectivity, for gas sensing is more challenging. In bio-sensors and BioFETs the electrolyte serves to keep undesired molecules out of the gate or channel regions. For gas sensors, on the other hand, exposing the channel to the ambient means it is exposed to any molecule which may come in its vicinity.

**Figure 11:**
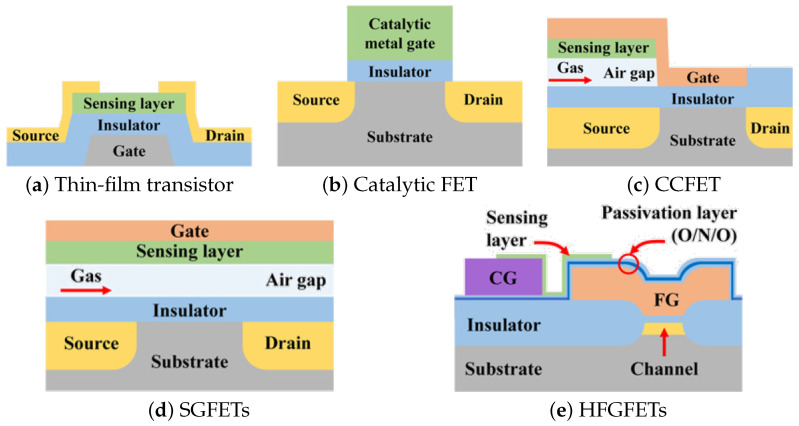
Schematic of different types of gas sensors which use the field effect of a FET for transduction. (Reprinted with permission from Hong et al. [118]).

A proper review of FET-based technologies for gas sensors is presented by Hong et al. in [118]. Here, we will provide only a general description of available technologies in order to understand how they relate to potential CMOS integration and, in particular, integration with digital logic transistors. The key to FET-based gas sensors is ensuring that the ambient gas, which should be detected, is able to control the conductivity in the channel by modifying the electric field through the gate oxide, effectively by manipulating the field effect. Access to the channel can be obtained through several configurations, including thin-film transistors, catalytic metal gate field-effect transistors (MGFETs), suspended gate field-effect transistors (SGFETs), capacitively-coupled field-effect transistors (CCFETs), or horizontal floating-gate field-effect transistors (HFGFETs), all of which are shown in Figure 11.

From the different types of FET-based gas sensing structures, the TFT is one which appears to be most promising and has been tested most extensively using 2D semiconductor materials [114,121]. The reason is that it is relatively easy to fabricate test devices, and the sensitive 2D film is the one which is directly exposed to the gas and acts as a FET channel layer. While this device seems promising for CMOS integration, the fact that it requires a back-gate is a concern. Incorporating a back-gate on a wafer-scale makes it difficult (or impossible) to control the electrostatics of each transistor’s gate independently. Therefore, for CMOS integration, once again, BEOL compatible solutions are being investigated [132,133,134,135,136,137]. However, BEOL integration comes with its own problems, as discussed in the chemiresistive section, in that the thermal budget is significantly reduced, the insulators are of lower quality, and mass production is very challenging.

The catalytic FET sensor works on the principle that the work function of the gate metal, such as palladium, platinum, or gold, changes when exposed to an increased concentration of certain gases. Most often, these are small gas molecules such as H_2_ [138] or CO [139]. The H_2_ adsorbs on the outer surface of the metal gate and dissociates into H atoms which then diffuse through the catalytic metal gate, moving downward towards its interface with the insulator. At the interface, a dipole layer is formed, changing the surface potential of the gate and shifting the threshold voltage V_t_ of the FET. This feature of the catalytic metal films is also used at the main sensing principle in Schottky-barrier gas sensors [140,141,142]. Furthermore, it should also be noted that these types of sensors are relatively easy to integrate with CMOS fabrication, albeit the inclusion of catalytic metals such as gold or platinum can be problematic. However, their major limitation is that they can only be used to detect very small molecules, preventing their broad applicability for gas sensing of hazardous air pollutants or for bio-sensing of VOCs in exhaled breath for medical applications.

Since the catalytic FET sensor is limited to detecting only small gas molecules which are able to diffuse through the metal layer, CCFET and SGFET structures have been proposed. These devices include an air gap between the gate insulator and sensing layer, whereby larger molecules are also accessible. The air gap, however, makes fabrication very difficult, often requiring flip-chip technology, which complicates potential scaling, mass production, and CMOS integration [143]. Alternatively, investigations are also underway into how one could introduce these structures after BEOL fabrication in a modified CMOS flow [144]. The horizontal floating-gate field-effect transistor was proposed to overcome the disadvantages of other FET-based sensors [145]. The HFGFET is fabricated using CMOS technology, and the process is similar to a conventional MOSFET. In this design, the sensing layer is independent of the transistor channel, as it should not contaminate the FET, meaning that there is reasonable potential for integration of 2D films as the sensing layer without disturbing the FEOL process flow. The presented implementation involves the use of SMO layers, such as SnO_x_ [145], ZnO [146], WO_3_ [147], or In_2_O_3_ [148] which need to be heated to elevated temperatures in the same way as SMO-based chemiresistive sensors.

## 4. Fabrication and Working Principle of 2D-Material-Based Gas Sensors

The semiconductor (or microelectronics) industry is clearly dominated by silicon, and the introduction of new applications and technologies on silicon is usually a stepping stone towards mass production and adoption in the market. Over the years, many materials have attempted to replace silicon, including materials with higher charge carrier mobility, such as germanium, and various group III-IV materials. However, none have been successful in commercialization on a broad scale and have only made breakthroughs in certain niche markets [22]. However, the continued scaling of silicon appears to have reached saturation and sub-3 nm channels pose significant challenges due to increased variability and reliability issues, but also due to the limited carrier mobility at these reduced scales [149,150]. This is precisely why it is expected that 2D semiconductor materials will be able to make a breakthrough in the coming years. Two-dimensional semiconductors are thermodynamically stable, even down to single atomic layers, and ideally, they come with inert, defect-free surfaces [151].

Beyond transistor scaling and digital logic, interest in 2D materials has intensified over the past years due to their potential usability in a wide range of applications. Due to the low dimensionality of these materials, they exhibit properties of relevance to several research fields from solid-state physics to low dimensional molecular chemistry. Therefore, even minor shifts in the chemical structure of the film’s surface can be felt in its bulk properties. This feature makes 2D materials ideal for catalysis [152,153,154,155] and sensing [93,156,157]. The range of application for gas sensing is immense and includes devices which are able to detect hazardous gases, organic vapors, and humidity: Devices frequently used for medical diagnostics, environmental monitoring, and safety and security [157]. TMDs including MoS_2_, MoTe_2_, WSe_2_, and SnS_2_ have already been widely studied for gas sensing in the FET configuration, primarily for the detection of nitrogen-containing compounds. In the chemiresistor configuration, a broader group of 2D materials have shown high potential for gas sensor development, including TMDs, boron nitride, black phosphorus, and MXenes [1,121]. The recent review of these materials by Wang et al. [121] goes into some detail discussing the potential of these films for gas sensing. However, they do not discuss the means or potential for their integration with CMOS fabrication or electronics, which is the main basis for this review. What makes 2D materials even more interesting for sensing is their potential to detect a broad range of disease biomarkers, both as a gas sensor of VOCs from exhaled breath, and from bodily excretions, such as urine, blood, tears, or saliva in the form of 2D-based bioFETs [158,159]. In general, there is great hope that 2D materials can revolutionize medical diagnostics and healthcare [160,161,162,163].

Nevertheless, while there are obvious theoretical benefits to future applications of 2D materials, there are still many challenges to overcome, specifically those related to the fabrication of devices based on these films. There are several methods which have been experimentally shown to produce reasonably defect-free films; however, the fabrication techniques which have the highest potential of CMOS-integration still suffer from several show-stopping problems. These are discussed in the next section.

### 4.1. Synthesis of 2D Materials

A thorough review of the means by which 2D materials are synthesized is provided by Knobloch in [164]. Here, we look at these processes from the perspective of their CMOS integration. The initial discovery of 2D materials for electronic applications was enabled by the mechanical exfoliation of single or few layers of graphene from a layered bulk crystal graphite [151]. While this process is not scalable and does not lend itself to CMOS integration, it is still frequently used in lab-based research into 2D materials and devices due to the simplicity of the process and the reasonable quality of films which can be achieved. The process involves thinning down a thick layer of a 2D crystal by placing it on adhesive tape. Subsequently, by frequently folding and unfolding the tape, increasingly fewer layers remain. These layers are then transferred to a wafer, typically SiO_2_-on-silicon [164,165]. While significant progress has been made to refine this process, mechanical exfoliation is still inherently a random process, whereby only a small fraction of the produced flakes are suitable for testing or device integration. Therefore, scalable processes, which are also CMOS-friendly and which produce high-quality 2D films, must be found if devices based on these are to truly compete with silicon on the semiconductor market. In this section, we briefly look at several potential means to synthesize 2D films, such as chemical vapor deposition (CVD), physical vapor deposition (PVD), molecular-beam epitaxy (MBE), and atomic layer deposition (ALD), and discuss their potential for use in a CMOS foundry for realizable devices.

#### 4.1.1. Chemical Vapor Deposition

**Figure 12:**
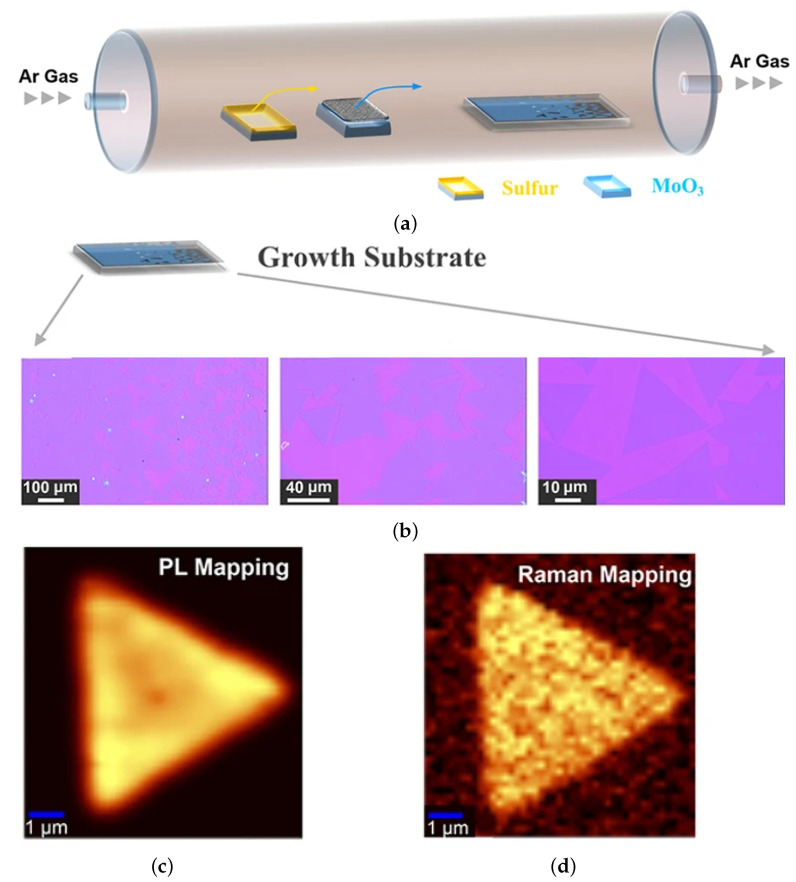
(**a**) Schematic of the CVD setup for the growth of ML MoS_2_. (**b**) Shows the optical images of the grown ML at different size scales. (**c**,**d**) show the photoluminescence and Raman intensity maps, respectively, of the MoS_2_ ML, grown on a SiO_2_/Si substrate. (Reprinted (adapted) with permission from Shi et al. [166], CC-BY 4.0).

CVD is probably the most widely studied means of depositing a film and is a staple of the microelectronics industry and of a CMOS foundry. It is a relatively simple bottom-up growth process which offers flexibility of metal precursor and a relatively fast growth rate. In Figure 12a, a typical CVD furnace for TMD growth is depicted, while in Figure 12b, the optical images of a grown monolayer (ML) is shown at different scales, with the typical triangular flakes quite visible when the resolution is sufficiently large. The triangular flakes are then further confirmed in Figure 12c and Figure 12d, where the photoluminescence and Raman intensity maps, respectively, are shown, from [166]. Unfortunately, the temperatures which are commonly required to grow 2D materials lie in the range of 600 °C to 1200 °C [167,168]. The typically required temperature is too high for BEOL integration, which requires temperatures in the 400 °C to 500 °C range. Some recent studies have worked to reduce the temperature, achieving minimal gains and managing to grow MoS_2_ films at 560 °C for 50 min [169] or WSe_2_ at 550 °C [170], albeit with slight modification to the typical CVD method.

It should be noted that several studies have examined the growth of graphene, hexagonal Boron Nitride (hBN), and TMDs using CVD. However, only a few studies have looked into growing black phosphorene with this method. This has to do with black phosphorus’ tendency to quickly oxidize in the presence of oxygen, requiring an oxygen-free growth environment. Furthermore, as can be seen from Figure 12, a precursor (e.g., chalgogen sulfur for MoS_2_) is required to grow the material; for phosphorene, this would be phosphine, a highly toxic material [171].

A variation on CVD, mainly metal-organic chemical vapor deposition (MOCVD), has also been used recently to grow 2D semiconductors [172]. This process uses gaseous metal–organic compounds as precursors instead of the solids in powder form used during CVD. This provides the advantage of temperature reduction down to a BEOL-compatible 450 °C, and the precise control over the partial pressures of all precursors grown in the chamber, which can now be introduced using mass flow controllers [173,174]. While this process has shown excellent yield and layer uniformity, it comes with a high density of trap states in the deposited films [175]. In fact, single-crystalline layers of 2D materials have, to date, not been successfully grown using MOCVD [164].

#### 4.1.2. Physical Vapor Deposition

**Figure 13:**
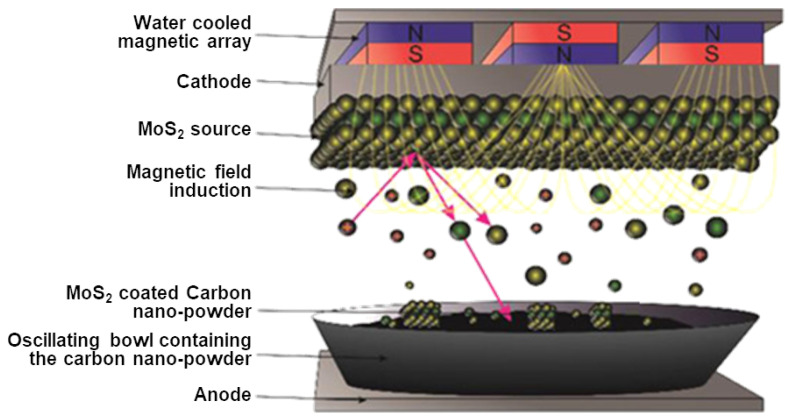
Magnetron sputter schematic of MoS_2_ deposition on carbon nano-powders. (Reprinted (adapted) with permission from Rowley-Neale et al. [180]; further permissions related to this material excerpted should be directed to the ACS).

A typical alternative to CVD in a CMOS foundry is PVD, which most often refers to thermal evaporation deposition or sputtering. PVD belongs to a family of synthesis processes which enable large-scale processing of 2D van der Waals (vdW) materials. There is no fundamental limit on the size or shape of the films which can be generated using this process, which has been used to produce thicker vdW films for decades [176,177], while the deposition of a few layers has also been recently demonstrated [178,179]. PVD also does not require the transfer of the grown material onto the desired substrate, as growth on any substrate is inherently possible. Figure 13 depicts a typical magnetron sputter MoS_2_ source which acts as a target [180].

The main principle behind sputtering is that a chunk of the target material is bombarded by ions from a gaseous plasma, whereby small sections of said material (down to single atoms) is sputtered off of the target. These sputtered particles then travel through the chamber, ultimately depositing onto the desired substrate. A magnetic source is often integrated with the target in order to confine the electrons to the regions close to the target’s surface. This type of sputtering is called magnetron sputtering, and it can be used to sputter any film, regardless of its melting temperature. Magnetron sputtering has been used to deposit both MoS_2_ [181,182] and WS_2_ films [183]. The major concern with sputtered 2D films is that the material which is deposited is polycrystalline and often sub-stoichiometric [181]. The high polycrystallinity leads to very low charge mobilities, in the order of 0.0136 cm^2^V^−1^s^−1^ and 0.0564 cm^2^V^−1^s^−1^ for bi-layer and five-layer MoS_2_, respectively [178]. The mobility can be brought up to about 10 cm^2^V^−1^s^−1^ after a high-temperature annealing step, which is still far below the mobilities achieved for CVD- or MBE-grown films [177]. Annealing the film at high temperatures would then negate the benefits that sputtering would initially provide for CMOS integration.

#### 4.1.3. Molecular-Beam Epitaxy

Molecular-beam epitaxy is a process by which epitaxial growth can be performed on a large scale in an ultra-high vacuum chamber with pressures in the sub 10^−10^ mbar range [184]. In MBE, the precursor molecules form a film on a heated crystalline substrate, which only provides the crystallographic information for the formation of the new film [164]. Since the substrate does not provide any catalytic surface effects, MBE results in a direct *in situ* growth of vertically-stacked heterostructures [185]. A typical MBE setup is shown in Figure 14 from [186], which consists of Knudsen effusion cells which provide the precursors by thermal evaporation in the form of a molecular beam. The substrate is located at the focal point of the effusion cells and is held in place with a holder. Monitoring the film crystallinity and growth rate is achieved using reflection high-energy electron diffraction (RHEED), while a residual gas analyzer (RGA) measures the partial pressure of the various gas species which are present in the chamber.

MBE is a very powerful tool for growing high-quality crystalline 2D films and has been extensively used to realize many films, including graphene, TMDs, and elemental 2D materials [187]. In addition, MBE has been applied to grow vertical and lateral vdW heterostructures [188]. There are, however, several difficulties in integrating MBE within a CMOS technology flow. The high vacuum requirements and high process sensitivity to small variations are a particular concern for mass production. Therefore, the technology remains principally a tool for studying the fundamental properties of various material systems [189]. Nevertheless, should these items be resolved and if the benefits for MBE integration in a CMOS foundry outweigh the costs, it is not unfeasible that we may see this integration in the coming years. In fact, there has already been some demonstrations of the benefit of MBE for multi-wafer vertical-cavity surface-emitting laser (VCSEL) fabrication, which may strengthen its push towards mass-production CMOS foundries [190,191].

#### 4.1.4. Atomic Layer Deposition

Atomic layer deposition is a method of thin film deposition which offers more control over film conformality and thickness than traditional CVD. Because it may be used to deposit technologically important oxides and nitrides, such as the gate oxide HfO_2_ and gate metal TiN, ALD has emerged as a fundamental technique in semiconductor processing for advanced nodes. This is primarily due to the self-limiting nature of the process. In simple terms, ALD requires at least two self-limiting steps, during which different gases are allowed to interact with the surface, in order to ultimately initiate the deposition of a single monolayer of a material. During each step, a surface catalytic reaction takes place, which ensures that the surface is covered with a specific precursor. This precursor then only reacts with the species which enters the chamber in the second step, thereby forming the desired film. In this way, ALD can be used to grow very precise thin films with excellent conformality and thickness control down to the angstrom level [192].

The ALD process also does not require very high temperatures, which means that it could be the solution for the BEOL CMOS integration of 2D materials [193]. There has already been a demonstration of the successful growth of ML and bulk MoS_2_ using ALD at 300 °C using MoCl_5_ and H_2_S precursors for Mo and S, respectively [194,195]. The major concern with ALD is that the deposition conditions, including the substrate material, have been shown to significantly impact the nucleation and growth of the films [196]. For example, plasma enhanced atomic layer deposition (PEALD) of WS_2_ on Al_2_O_3_ was shown to be very reactive, producing small grains, while using SiO_2_ as the substrate was less reactive and larger grains were produced [196]. In each case, the crystallinity of the film was significantly lower than when using CVD or MBE processes, which is why a post-ALD annealing step is often required in order to improve the crystallinity and the material parameters, similar to sputtering. However, this step usually requires very high temperatures, up to 800 °C or 900 °C, making it impossible to integrate this process with the BEOL currently [197].

**Figure 15:**
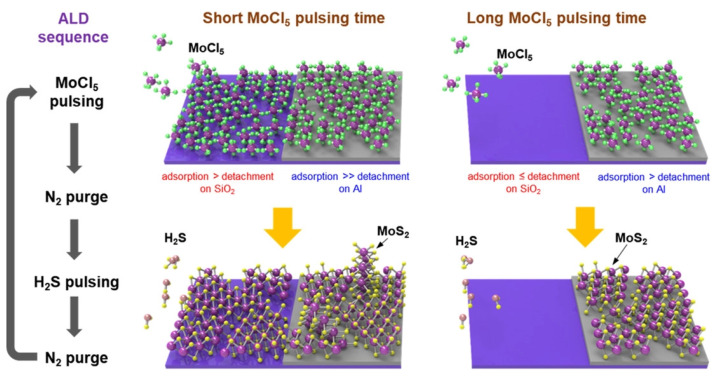
Schematic of the area-selective ALD process of MoS_2_ showing the impact of the MoCl_5_ pulse duration on the deposition of MoS_2_ on different surfaces (SiO_2_ and Al). (Reprinted with permission from Ahn et al. [198]).

An additional concern with the integration of 2D materials with CMOS technology is the difficulty in patterning the films, which will be critically damaged if exposed to plasma etching. A recent study by Ahn et al. [198] showed that it was possible to simultaneously deposit and etch MoS_2_ layers using MoCl_5_ and H_2_S precursors at 400 °C. Essentially, the authors show a selective deposition process, whereby the Mo-precursor MoCl_5_ would not adsorb onto the SiO_2_ surface, while adsorbing onto the surface areas which were covered by aluminum, even after 400 ALD cycles, shown in Figure 15. This only happened when the MoCl_5_ pulsing time was long, at 5 seconds; when the pulsing time was set to 1 second, both SiO_2_ and aluminum surfaces were fully covered, as expected with an ALD process. This finding could lead investigations towards a plausible method to pattern these films in a process which is compatible with the CMOS foundry.

Ultimately, even though the ALD technique has successfully been applied to grow several 2D semiconductors, the need to perform a post-ALD anneal at high temperature is a hindrance to CMOS integration. If an alternative annealing method is found, which does not require high temperatures and which is BEOL-conforming, then ALD could find itself as the foremost enabling technology which brings 2D materials inside the CMOS foundry. Furthermore, ALD involves complex chemistries and many experiments must be performed in order to find the proper precursors and the ideal chamber conditions for each of these to grow monolayers of a particular material. This means that, for a new material, an entirely new chemistry must first be identified and then fine-tuned before it can be applied to produce usable films.

### 4.2. Gas Sensing Principles of 2D Materials

In the previous sections, we have described the capabilities exhibited by 2D materials for several applications in very broad terms, while considering the limitations and potential of CMOS integration. In the section which follows, Section 5, we will dive into recent demonstrations of the sensing capabilities of many relevant 2D semiconductors. However, before we look at the experimental achievements of 2D-based sensors, in this section, it is important to describe what is so special about 2D materials that makes them highly promising for the future of gas sensing and why so much effort is being devoted into their efficient fabrication and realization. As mentioned previously, much of this has to do with the potential of low-temperature performance, eliminating the need for microheaters in MEMS-CMOS integration, and the potential for full CMOS integration, albeit more work still needs to be performed to achieve these goals. When we looked into the different semiconductor-based gas sensor types, we mainly analyzed chemiresistors and FET-based sensors, cf. Section 3, because of their high potential for CMOS integration in comparison to alternate solutions, such as optical sensors. These types of sensors utilize either the resistive change of a film or the field effect of a transistor through changes on the film’s surface as a consequence of its direct interaction with ambient gas molecules.

As discussed in Section 2.3, the main sensing mechanism of SMO sensors is through the surface adsorption of oxygen ions, such as O^2−^, O^−^, and O_2_^−^ [199]. The presence of the oxygen ions on the surface create a depletion region, which is then reduced when these ions react with gas molecules of interest. Therein lies the core of the sensing mechanism for SMOs: For n-type sensing materials (e.g., SnO_2_, ZnO, TiO_2_) the resistance will decrease or increase, depending on if it is exposed to reducing or oxidizing gases, respectively. The inverse is the case when a p-type SMO material is used (e.g., CuO, NiO, Cr_2_O_3_). For gas sensors based on 2D materials, however, the process does not require oxygen adsorption and the mechanism mainly follows the charge-transfer process [121]. This means that the sensing film will act as a donor or acceptor of charges from the adsorbed gas molecule during the charge transfer procedure. Since different gases are able to exchange charges with the 2D film, it is the amount of charge that is exchanged, leading to changes in the conductive behavior of the film, that can be used to classify the specificity of the gas sensor.

**Figure 16:**
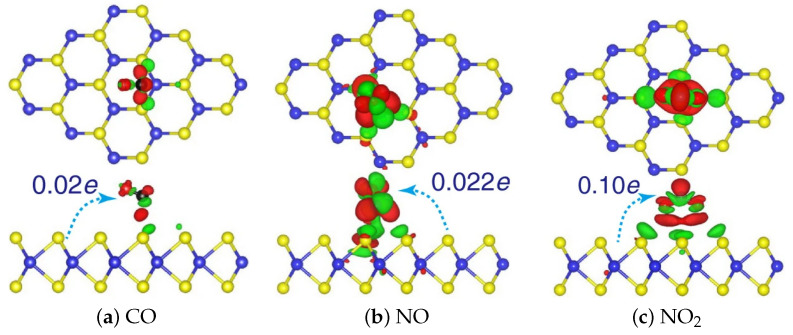
Charge density difference plots for adsorption of (**a**) CO, (**b**) NO, and (**c**) NO_2_ on ML MoS_2_. The red (green) distributions correspond to charge accumulation (depletion). (Reprinted with permission from Yue et al. [200], CC-BY 2.0).

A schematic diagram of the charge transfer mechanism which occurs when an n-type 2D semiconductor, such as MoS_2_, is exposed to several hazardous gases (i.e., CO, NO, and NO_2_) is provided in Figure 16, from [200]. Since the MoS_2_ monolayer is n-type, when it is exposed to CO, NO, or NO_2_, the electron charge is transferred from the film to the gas, meaning that an effective positive charge is contributed to the sensing film by the physically absorbed molecule, increasing its resistance. The absorption of NH_3_, on the other hand, will cause for a charge to be donated to the MoS_2_ sensing film, resulting in a resistance decrease.

**Figure 17:**
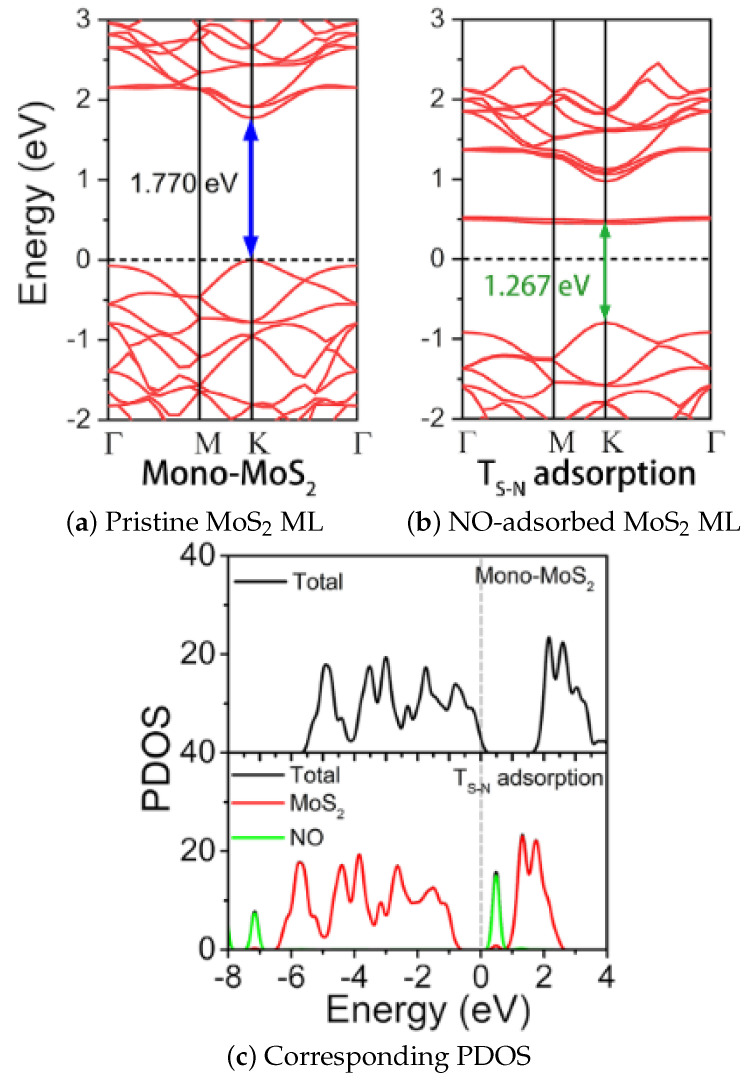
Calculated band structures and corresponding PDOS of (**a**) pristine ML MoS_2_ and (**b**) NO-adsorbed ML MoS_2_. In (**c**), the corresponding PDOS is provided. The Fermi energy is set at 0 eV. (Reprinted (adapted) with permission from Wang et al. [201], CC-BY 4.0).

It should also be noted that the adsorption of gas molecules on the ML MoS_2_ surface results in a change in its band structure, depicted in Figure 17a,b in the case of NO adsorption, from [201]. The adsorbed NO introduces additional energy states at 0.5 eV, giving rise to a significant upward shift in the Fermi level. The partial density of states (PDOS) plots Figure 17c show that the there are additional states, which are primarily induced by the adsorbed NO gas (i.e., green line in Figure 17c).

Similar studies to the one described for ML MoS_2_ have also been performed for p-type 2D semiconductors [202]. Therein, the authors look at how several relevant gas molecules, such as CO, H_2_O, NH_3_, O_2_, NO, and NO_2_ impact the electrical properties of the p-type WSe_2_ ML. This p-type semiconductor shows the opposite behavior to what was observed with MoS_2_. Mainly, CO, NO, and NO_2_ adsorption causes a charge consumption at the WSe_2_ surface, while NH_3_ induces charge accumulation. Currently, there are two main ways that gas sensing can proceed in the case of chemiresistive or FET-based sensors which rely on surface chemical reactions: Reaction with surface-adsorbed oxygen at high temperatures (i.e., SMO sensors) and charge-transfer (i.e., 2D semiconductor sensors). Therefore, the implied advantage of using 2D materials is that an oxygen-rich environment is not a precondition for a sensing response. It also suggests that sensing with 2D materials will be less sensitive to other changes in the ambient environment, such as O_2_ content or relative humidity.

It should be noted that the above discussion primarily concentrates on the physical absorption of gas molecules on a pristine surface of a 2D ML. However, as we have discussed in Section 4.1, the synthesized film can often be polycrystalline or contain many defects, edges, and grain boundaries, where gas molecules can often adsorb [203,204]. Understanding the interaction between a gas molecule and a broad variety of defects in the MoS_2_ structure is a stepping stone towards a thorough understanding of its behavior at grain boundaries and edges. In gas sensor applications, adsorbed air molecules can hinder the selective detection of VOCs which are a set of organic gases playing a key role in air pollution detection and medical diagnostics [205]. While the sensitivity in a dry environment would support excitement about MoS_2_, the authors in [205] found that humidity had a large effect on the resistive response. Furthermore, they note that at relative humidity above 60% this response increases drastically. This demands a thorough analysis, because the dependence of humidity on the sensitivity is the biggest challenge for sensor integration into real-world medical diagnostics, especially for breath analysis, which contains at least 80% humidity [206].

Many researchers have applied *ab initio* calculations to study surface physisorption of several different gas molecules on a pristine, non-defected MoS_2_ surface [207,208]. The interactions described there depend mostly on vdW interactions between the gas molecule and the MoS_2_ film. However, these weak forces alone were unable to explain many observed changes in the film’s electrical properties under varying ambient conditions [207,208]. These changes are proposed to be induced by the interactions between the gas molecules and point defects in the 2D semiconductor. It is currently accepted that six types of point defects are observable in CVD-grown ML MoS_2_: Sulfur monovacancy (V_S_), sulfur divacancy (V_S_2__), vacancy complex of Mo and nearby three sulfur (V_MoS_3__), vacancy complex of Mo nearby three disulfur pairs (V_MoS_6__), and antisite defects where a Mo atom substitutes a S_2_ column (Mo_S_2__) or a S_2_ column substitutes a Mo atom (S2_Mo_). Many of these are noted at the edges and grain boundaries, while V_S_ can readily appear also in the surface of a crystalline film [209]. The stability of different point defects has been explored through their formation energies. In the entire range of sulfur’s chemical potential and of all the listed types of point defects in MoS_2_, V_S_ and Mo vacancies were found to have a very low (2.35 eV) and a very high (8.02 eV) formation energy in S-rich and Mo-rich conditions, respectively [210]. Therefore, it is not surprising that the most commonly observed defect is V_S_ and that this is usually assumed to be the main adsorption site for gas molecules [204,208,211]. Several studies have taken to first principles simulations in order to understand the interactions between gas molecules and differently defected MoS_2_ surfaces [207,212,213,214,215].

## 5. Two-Dimensional-Material-Based Gas Sensing Films

**Figure 18:**
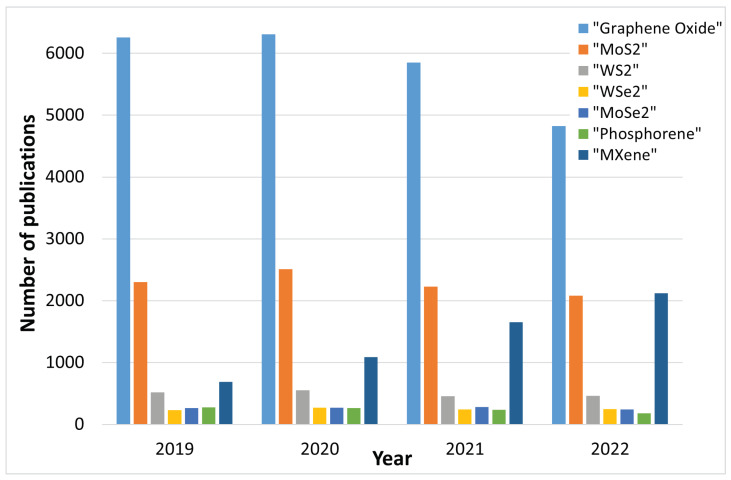
Number of published manuscripts indexed by Scopus since 2019 with “Graphene Oxide”/“rGO”, “MoS2”/“Molybdenum Disulfide”, “WS2”/“Tungsten Disulfide”, “WSe2”/“Tungsten Diselenide”, “MoSe2”/“Molybdenum Diselenide”, “phosphorene”/“Exfoliated phosphorus”, and “MXene” in the title. (Source: Scopus, 28 September 2022).

The change in the conductivity of graphene and other 2D materials as a result of a changing make-up of the ambient environment is already proven, and publications in this direction are plentiful. Even as early as 2007, the Nobel laureates for graphene’s fabrication and characterization (i.e., Geim and Novoselov [151]) described and reported on graphene’s changing electrical properties due to exposure to NO_2_ and NH_3_ [216]. Specifically, they showed that the adsorbed molecules increased the charge carrier density of graphene, with paramagnetic molecules such as NO_2_ acting as an electron dopant. However, due to graphene’s lack of a band gap, researchers have been unable to develop a functional digital logic FET. As a result, other possible 2D materials have gained momentum, which exhibit the presence of a reasonably large band gap while also having tremendous potential for concurrent FET, optical, and sensing applications. This section discusses these materials, which include graphene-based films such as graphene oxide and reduced graphene oxide, TMDs such as MoS_2_ and WS_2_, phosphorene, and MXenes such as Ti_3_C_2_Ti_x_, for gas sensing applications. In Figure 18, the total number of published articles with the words “Graphene Oxide” or “rGO”, “MoS2” or “Molybdenum Disulfide”, “WS2” or “Tungsten Disulfide”, “WSe2” or “Tungsten Diselenide”, “MoSe2” or “Molybdenum Diselenide”, “Phosphorene” or “Exfoliated phosphorus”, or “MXene”—which summarize the most actively investigated 2D materials—in the title is reported. We note that while graphene oxide (which includes reduced graphene oxide) and MoS_2_ are still leading the conversation, the number of articles on these topics is slowly plateauing or declining. At the same time, interest in MXenes has increased significantly in the last few years.

### 5.1. Graphene Oxide and Reduced Graphene Oxide

As already discussed earlier, considerable attention has been attracted by graphene for various applications. This is most likely due to it being the breakthrough 2D material, which was fabricated and investigated at its monolayer the earliest. Graphene was shown to be particularly interesting for gas sensing applications due to its extremely high specific surface area, which interacts with certain gases to change the film’s physical properties [217]. However, its near-zero band gap is a significant hurdle for its broad applicability in transistors and sensing devices. Therefore, many researchers began investigating functionalized or decorated graphene using graphene oxide (GO) [218,219]. Strong oxidizers can be used to treat graphite in order to separate the graphite from the GO flakes. Through subsequent graphite exfoliation, only the GO flakes remain [220]. The modified Hummers’ method [221,222,223] is the primary means applied to generate wider GO flakes with a lower number of defects, with the hopes of increasing the production yield.

Graphene oxide (GO) is an analog of graphene with many functional groups which ensure that the physical and chemical properties of the film are significantly different to that of graphene [218]. Reduced graphene oxide (rGO) is synthesized from GO and is often treated as graphene in publications and discussions. However, a key difference is that rGO sheets, like GO, are inexpensive and easy to prepare while having the benefit of a large surface-to-volume ratio. Reduced graphene oxide can also be tailored to exhibit properties from insulating (i.e., near-GO) to near-metallic (i.e., fully reduced GO will theoretically produce graphene), depending on the process used to reduce GO.

GO flakes have been readily applied for the fabrication of an impedance sensor for the detection of relative humidity (RH) [224]. The real and imaginary components of the GO’s dielectric constant are used in these investigations as a sensing signal [225]. Molecular surface adsorption causes the dielectric constant to fluctuate, which in-turn causes the frequency response to alter, thereby shifting the resonant frequency which can be detected. For fast and precise RH detection, this mechanism has been recently used in a piezoelectric micromachined ultrasonic transducer, combined with a GO sensing layer, as shown in Figure 19 from [226].

Recent implementation of GO sheets for humidity detection are based on porous laser-induced graphene electrodes to ensure good electrical properties and high mechanical stability [227,228,229]. The main sensing principle of these flexible devices is capacitive, wherein the capacitance of the GO sensing layer changes when varying the humidity from 11% to 97% at a frequency range from 20 Hz to 10 kHz [227].

**Figure 20:**
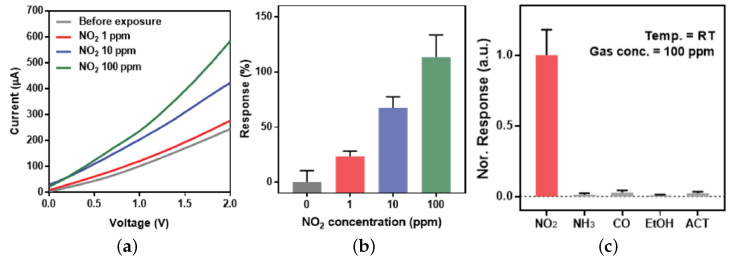
NO_2_ detection using a paper-based rGO–chitosan composite sensor from [236]. (**a**) Current–voltage curves of the sensor; (**b**) Relative response according to NO_2_ concentration; (**c**) Normalized specificity of the sensor towards NO_2_ compared to NH_3_, CO, Ethanol (EtOH), and Acetone (ACO). (Reprinted (adapted) with permission from Park et al. [236]).

With increasing concentrations of RH in the ambient, the resistivities of conductometric gas sensors based on GO flakes have shown minimal variation [220,230]. This is in stark contrast to SMOs, whose sensitivity greatly depends on the ambient humidity and is a highly positive development when discussing the potential applicability of sensing exhaled breath in early medical diagnostics and disease detection. The main obstacle for the development of an exhaled breath sensor for real-world medical applications is the effect of RH on the sensitivity. This is a particular concern for breath analysis, since exhaled breath comprises at least 80% humidity [206]. However, in a recent GO-based study of NO_2_ sensing, when the RH was increased from 50% to 75%, concentrations which are very important for breath detection, it was demonstrated that the relative response did not change at all [231], providing some optimism for the potential future use of these films in this important field. The functional oxygen groups on the surface of GO films are thought to be the primary reason behind GO’s ability to detect NO_2_ at low concentrations, even at room temperature, mainly because graphene and rGO do not show high responses to NO_2_ [232,233]. At the same time, graphene oxide is being applied in composite layers in order to increase the sensitivity or selectivity of the sensing behavior towards a desired gas [234,235,236]. A recent study shows the applicability of rGO–chitosan composite layers for flexible and disposable paper-based NO_2_ sensors [236], with results given in Figure 20.

Surface functionalization techniques have readily been applied for the fabrication of SMO-based sensors. This is a common method to engineer additional sensitivity and selectivity and improve the overall performance of chemiresistive gas sensors. Researchers have been investigating the potential of functionalizing and micromachining the GO surface to improve its sensing performance and, in particular, to ensure selectivity/specificity towards a particular gas molecule [220]. For instance, to improve specific SO_2_ and NH_3_ detection at room temperature, specially tailored GO flakes have been fabricated, as described in [237] and [238], respectively.

Graphene oxide exhibits a very high resistivity, while its permittivity is often affected by the ambient, which is why so many capacitive sensors are designed with GO as the sensing layer. In order to partially recover the highly conductive behavior of pristine graphene films for chemiresistive sensors, the GO layers are typically reduced, generating rGO flakes [219].

**Figure 21:**
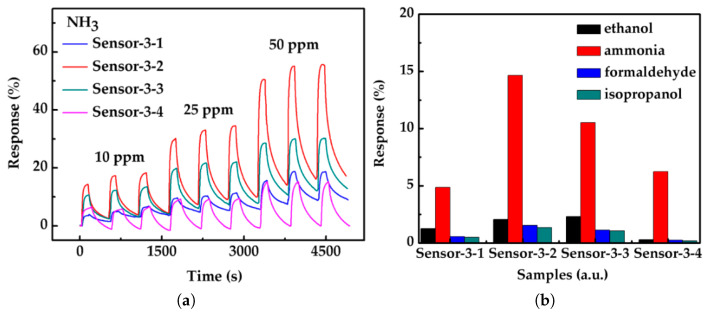
(**a**) Sensing response for the differently-reduced hybrid rGO–graphene sensing films, when exposed to three different concentrations of ammonia. (**b**) A study on the selectivity of the rGO–graphene hybrid sensor towards ammonia, when compared to isopropanol, formaldehyde, and ethanol at 10 ppm. (Reprinted with permission from Wang et al. [245], CC-BY 4.0).

Because of the increased conductivity of rGO films and due to the presence of hydroxyl groups on their surface, which facilitate adsorption, these materials have been extensively researched for their potential use in gas sensing devices [121,220]. The degree of reduction can also be tuned and adjusted to tailor the film’s applicability towards a specific use-case. Many important gases and compounds can be detected using these films, even at ambient temperature. For example, rGO flakes have been used for the detection of NH_3_ at concentrations ranging from 5 ppb to 100 ppm [239], NO_2_ for concentrations at ppb levels [240,241], hydrogen at ppm levels [242,243], and even chemical warfare agents down to the ppb level [244]. Figure 21 depicts the outcomes of recent research by Wang et al. [245] to develop a highly selective ammonia (NH_3_) sensor based on a hybrid rGO–graphene film. The four sensors under study therein are rGO–graphene hybrids, with varying reduction times being 0, 10, 20, and 40 minutes for the generation of the rGO layer of sensors labeled as “sensor-3-1”, “sensor-3-2”, “sensor-3-3”, and “sensor-3-4”, respectively [245]. The results demonstrate substantial selectivity towards NH_3_ for all tested reduction times, while the sample with the shortest non-zero reduction time (i.e., 10 min) exhibits the highest sensitivity. The synthesis of very sensitive and precise gas sensors has also shown promise, when the sensing capabilities of rGO films and SMO nanostructures are combined, albeit with a notable increase in fabrication complexity [246,247].

The functionalization of the rGO films and the creation of rGO composites with other semiconductor films has been utilized recently in order to increase the sensitivity and specificity towards a target gas molecule, most often nitrogen dioxide (NO_2_) [248,249,250]. Farea et al. in [251], used polymerization on GO to generate a polypyrrole–graphene oxide composite film as a cost-effective room temperature CO sensor. The authors observed short response and recovery times with excellent repeatability and long-term stability. Nevertheless, increasing the RH resulted in a severely degraded sensitivity response [251]. Chu et al. in [250], reduced a porous GO film using a hydrothermal process at 180 °C to design a sensor which is able to detect NO_2_ down to 12 ppm, while claiming a theoretical limit of detection down to 0.66 ppb [252]. Yang et al. in [249], used a three-dimensional structure composed of MoS_2_/rGO nanosheet/graphene quantum dot layers to design NO_2_ sensors with a detection limit down to 5 ppm and a sensitivity towards NO_2_, which is ten times higher than towards other gases. Finally, the work by Umar et al. in [248] concerns an assembled isonicotinamide–rGO nanocomposite sensor for room temperature NO_2_ detection, showing a limit of detection down to 1 ppm and a linear response in the range of 1 ppm to 30 ppm. Remarkably, the device shows minimal drift over a period of 30 days.

**Figure 22:**
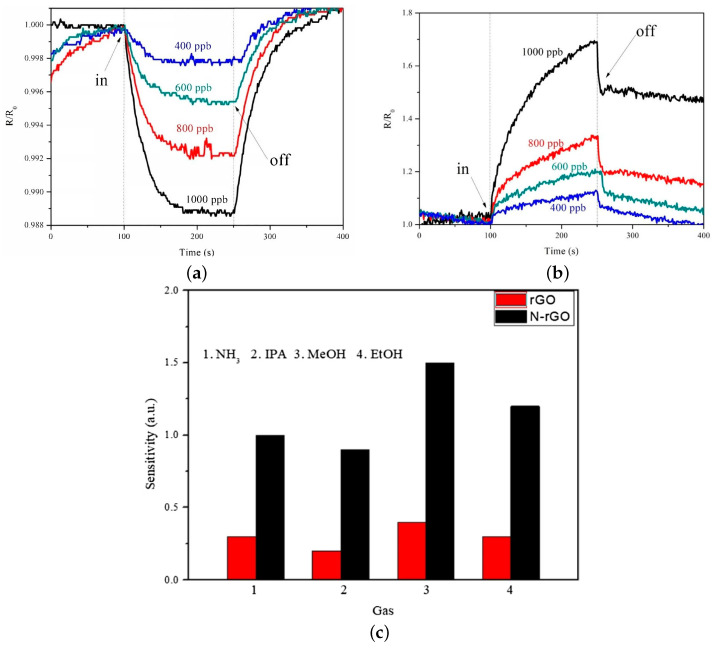
The relative resistance (R/R_0_) in various concentrations of 400–1000 ppb NO gas using (**a**) pristine rGO and (**b**) N-doped rGO. In (**c**), the response to other VOCs is given for pristine and N-doped (N-rGO) rGO, including NH_3_, isopropanol (IPA), ethanol (EtOH), and methanol (MeOH). (Reprinted with permission from Chang et al. [255], CC-BY 4.0).

Recent studies also show the application of rGO in combination with SMO films for targeted molecular detection. Ag-functionalized Fe_3_O_4_–rGO composites were presented with an improved sensitivity towards ammonia [253], while Cd-functionalized SnO_2_–rGO composites show an improved detection of carbon monoxide (CO) [254]. Other types of doping of rGO films have also been studied, such as nitrogen (N) doping for the detection of NO [255]. The authors show that the N-doped films exhibit an enhanced sensitivity to several gases, including NO, when compared to the pristine rGO alternative, shown in Figure 22. Furthermore, SO_2_ detection was shown to be possible by combining the SMO TiO_2_ with rGO in a buried-gate FET device [256]. The authors observed an almost three-fold increase in the sensitivity of a TiO_2_–rGO sensor to detect 20 ppm SO_2_ at room temperature compared to the result achieved by a SnO_2_ sensor for 500 ppt CO at 220 °C. The combination of rGO with the SMO ZnO appears to improve the sensitivity towards ozone (O_3_) [257]. The lower limit of detection of the composite film was shown to be ten times lower than when using a pure ZnO sensor.

### 5.2. Transition Metal Dichalcogenides

**Figure 23:**
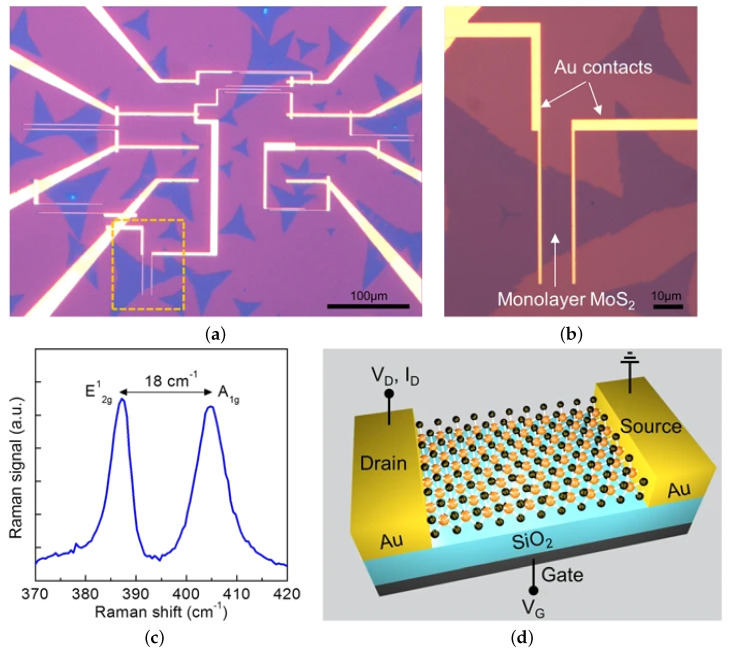
CVD-fabricated monolayer MoS_2_ FETs. (**a**) Image of the monolayer MoS_2_-based FET. (**b**) Single MoS_2_ flake with gold contacts on either side. (**c**) Raman spectrum of the fabricated film. (**d**) Typical 3D representation of a back-gated monolayer MoS_2_ FET on a SiO_2_ insulator. The substrate below the insulator is usually heavily doped to ensure sufficient control of the channel from the back gate. (Reprinted with permission from Ahn et al. [268], CC-BY 4.0).

Research on 2D transition metal dichalcogenides (TMDs) has been dramatically increased during the last decade and, in that time, many research groups have demonstrated working transistors with these films [136,258,259,260,261,262,263]. Among TMDs, molybdenum disulfide (MoS_2_) [22,259,263] and tungsten sulfide (WS_2_) [264] are of particular interest for transistors, since they are naturally occurring layered crystals, are robust and relatively abundant, and present a wide band gap; nevertheless, many other TMDs are also readily being investigated [132,260,265]. One of the main issues with 2D TMD semiconductor FETs is finding a fitting insulator, whether in the top-gated or back-gated configuration, where the interface defect concentration is minimal and does not negatively impact transistor operation or its long-term reliability [21,266,267]. It should be noted that the back-gated design, shown in Figure 23 for a CVD-grown MoS_2_-based FET is a desirable geometry for sensing applications, since it provides a direct interface between the sensing film and the changing ambient. Furthermore, a back gate can be used to control the electrostatics in the channel layer of the back-gated FET [268]. However, the back-gated configuration usually means that the entire wafer is set to the same gate bias, making it difficult to provide each transistor or FET-based sensor an independent gate potential.

MoS_2_ and WS_2_, in their bulk forms, are made up of several S–Mo–S or S–W–S planes, respectively, connected to each other through van der Waals forces. Researchers have demonstrated that these films can be synthesized using CVD [269,270], while they can also be exfoliated down to a ML thickness [271]. The bulk forms of MoS_2_ and WS_2_ films show indirect semiconductor performance, with band gaps of around 1.2 eV and 1 eV, respectively. The ML alternatives of these materials, however, are direct semiconductors and exhibit band gaps of 1.8 eV and 2 eV, respectively [272].

MoS_2_ has already been demonstrated to respond quite strongly to the presence of many gases of interest, even at room temperature, e.g., H_2_ [273], O_2_ [274], NO [275,276], NO_2_ [276,277], NH_3_ [278], H_2_S [279], humidity [280], and many more. MoS_2_ is shown to readily react and change its conductive behavior due to a change in humidity or oxygen concentration in the ambient air, meaning that hydroxil groups and oxygen readily adsorb on its surface. This may be a hindrance in the future wide application of MoS_2_ in microelectronic devices and sensors, as the presence of humidity and oxygen is unavoidable, especially when it comes to selective exhaled breath detection [206]. Furthermore, polycristalline MoS_2_ layers have been shown to exhibit high defect concentrations on their edges and at the grain boundaries, where gas molecules can frequently adsorb [203,204]. It should be noted that theoretical studies on the potential of MoS_2_ for the detection of a wide variety of gas molecules are abound [281,282,283,284,285]. However, here, we concentrate primarily on achievements made in gas sensing using fabricated devices in a lab environment to assess their potential for CMOS foundry integration.

**Figure 24:**
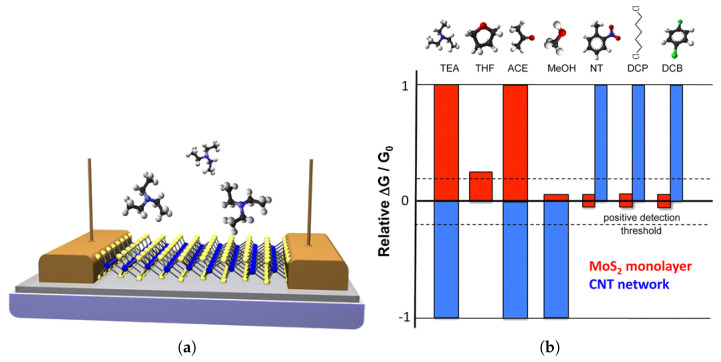
(**a**) Three-dimensional representation of a ML MoS_2_ resistor, while being exposed to a triethylamine (TEA) molecule. The sensing film is deposited on a SiO_2_-on-Si wafer and is electrically contacted with Au pads. (**b**) Relative change in the conductivity of the ML film from (**a**), and CNT for comparison, after exposure to several molecules, including triethylamine (TEA), tetrahydrofuran (THF), acetone, methanol, nitrotoluene (NT), 1,5-dichloropentane (DCP), and 1,4-dichlorobenzene (DCB). (Reprinted with permission from (**a**) Perkins et al. [288] and (**b**) Wang et al. [245], CC-BY 4.0).

Recent research has confirmed the possibility of employing TMDs for chemical sensing at ambient temperature. For the detection of NO_2_, recent research has demonstrated the excellent sensitivity and specificity of ML MoS_2_ films, when exposed to an ambient which includes H_2_, H_2_S, NH_3_ [286], and C_2_H_5_OH [287]. Recent studies have further confirmed the possibility of employing TMD films for chemical sensing at room temperature [68]. Monolayer MoS_2_ on a SiO_2_-on-Si substrate has recently been applied towards the selective detection of triethylamine (TEA) down to few ppb, when compared to its response against many other analytes [288], c.f. Figure 24. Many other TMDs have also already demonstrated their ability to sense a broad variety of ambient gases, including humidity, NO, NO_2_, NH_3_, H_2_, CO, and many more [68,220,280]. It is crucial to highlight that the pristine MoS_2_ surface does not appear to support broadly applicable and highly sensitive gas sensor applications since it does not provide strong-enough adsorption for non-polar gas molecules (such as CO_2_ and CH_4_). The charge exchange between MoS_2_ vacancy defect sites and a gas molecule of interest, however, was shown to provide significant changes to the resistive behavior of the film [289]. These defects are most frequently a sulfur (i.e., in MS_x_ films) or selenide (i.e., in MSe_x_ films) vacancy [210].

Similar to the studies related to graphene films, SMO-decorated MoS_2_ films have recently shown improved sensing performance towards several gases. In [290], the authors note an improved performance towards the detection of CO, NH_3_, and H_2_ after decorating MoS_2_ with SnO_2_ nanofibers. In addition, the MoS_2_–SnO_2_ sensors are able to operate at lower temperatures compared with the bare SnO_2_ sensors for SO_2_ detection at a concentration in the 1–10 ppm range [290]. Ultimately, they managed to fabricate an MoS_2_–SnO_2_ composite sensor which operates at 100 °C and exhibits a sensitivity (R_gas_/R_air_) of about 10.

**Figure 25:**
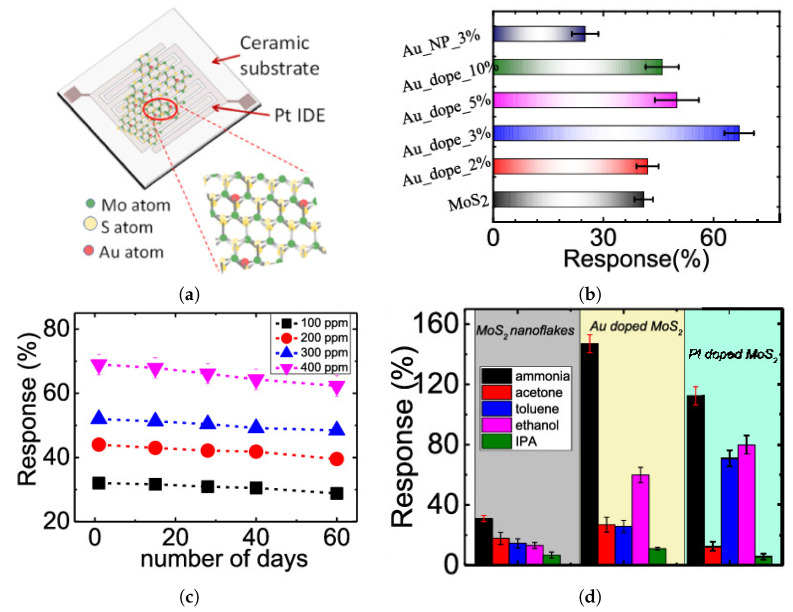
(**a**) Gas sensing device presented by Burman et al. [291]; (**b**) the response of samples with varying Au-doping concentrations in 400 ppm NH_3_ at 90 °C; (**c**) the stability of the sensors for two months under different NH_3_ concentrations; and (**d**) the selectivity of undoped, Au-doped, and Pt-doped MoS_2_ towards several VOCs. (Reprinted (adapted) with permission from Burman et al. [291]).

While the high sensitivity of TMD films has generally been proven experimentally and theoretically, recent research has turned increasingly towards improving selectivity. A general problem with chemiresistive gas sensors is the difficulty with which it can be known, which specific gas molecule has caused a particular measured change in resistance. The main method applied to introduce selectivity is through substitutional doping [291]. For MoS_2_, this entails replacing a sulfur atom with a metal atom, such as gold, which has shown to increase the sensitivity towards ammonia by 150%, while using platinum increased the sensitivity towards humidity by up to 60,000% [291]. The work by Burman et al. in [291] also shows that the 3% Au-doped MoS_2_ experiences the highest response towards ammonia, noting that increasing the dopant concentration further had a negative effect on the response. The results from this study are provided in Figure 25.

These results have been confirmed by *ab initio* studies which show that Au-doped and Au–Ag co-doped MoS_2_ films tend to increase sensitivities towards ambient NO and NO_2_ gases [276]. A similar study on indium selenide (InSe) showed that gold-modified 2D nanosheets of this material resulted in a significant increase in the sensitivity towards NO_2_ and NH_3_ [292]. The authors therein further discussed the potential to use light-illumination as a means to tune the selectivity towards desired molecules. In a bright ambient, the selectivity towards NO_2_ was increased ten-fold when compared to dark conditions, while the response to NH_3_ increased under dark conditions. The potential of doping tin sulfide (SnS_2_) MLs with platinum for gas sensing applications was also recently studied using *ab initio* techniques [293]. The authors appear confident that platinum doping can improve the sensitivity of these monolayers sufficiently enough towards small nitrogen-containing molecules (NO, NO_2_, and NH_3_) to make them a viable candidate for future gas sensors. Further examples of TMDs MLs which have been studied for their potential for gas sensor applications by substitutional doping include tantalum sulfide (TaS_2_) [294] and molybdenum telluride (MoTe_2_) [295].

The substitutional doping of TMD monolayers most often proceeds by populating an already existing vacancy, most often a sulfur or selenide vacancy, which is created as a result of the material synthesis. In the MoS_2_ example, sulfur vacancies can readily appear during fabrication or exfoliation [210,296]. These vacancies then serve as favorable adsorption sites for the metal dopant atoms [291]. Because these vacancies form during the exfoliation process, ref. [291] proposes a method to replace these vacancies with a desired metal atom directly during exfoliation. However, there is still a long way to go before mass production of such films can be realized. One recently studied alternative to vacancy doping is edge activation [297]. Essentially, TMD monolayers deposit as flakes and the edges of these flakes may contain several vacancies or dangling bonds, which can then be used as adsorption sites for metal atoms or gas molecules directly [297].

It should be noted that the vacancies can also serve as gas molecule adsorption sites, as shown in recent experimental and first-principles studies by Kumar et al. [271] and Jasmine et al. [298], respectively. Kumar et al. [271] specifically looked at NO_2_ adsorption. They noted a MoS_2_–NO_2_ adsorption complex, where the sulfur monovacancy acts as a singly ionized acceptor at 0.54 eV above the valence band. They also note a peak sensitivity for operation at 100 °C, which is a significant improvement over the requirement of SMO-based sensors. While room temperature operation is still desired, room temperature operation makes it difficult to design reusable sensors, since removing the adsorbate after a sensing event is not straight-forward. For operation at slightly elevated temperatures (i.e., near 100 °C), returning to room temperature should assist in the desorption of the adsorbed molecules. An alternative, already discussed previously, is UV illumination to assist in removing a surface-adsorbed molecule.

As can be observed, this section has primarily summarized the progress made in MoS_2_-based gas sensors. This was chosen, because MoS_2_ appears to be the 2D semiconductor, which is the closest to being realized with digital transistor devices and is one which has been heavily studied for gas sensor applications [22]. Nevertheless, at the beginning of this section, we also mentioned the potential that WS_2_ shows. In fact, gas sensing studies with WS_2_ appear to very closely follow the achievements made with MoS_2_. WS_2_ has shown a reasonably high sensitivity towards the detection of several relevant gases, including NO_2_ [299,300,301,302], NH_3_ [278,303], H_2_S [301,304], and CO [264]. Similar to MoS_2_, doping with metal atoms has been used to achieve an improved sensitivity towards particular gas molecules for enhanced selectivity. An example is using Au-decorated WS_2_ nanosheets for selective CO [264,305] or humidity [306] sensing. The edge states of WS_2_ flakes have also been explored for enhanced gas sensing and show increased selectivity towards NO_2_ [307]. More recently, ruthenium was also used to dope WS_2_ nanosheets in order to achieve an improved performance towards CO [308]. The study in [308] shows that Ru-doping increases the sensitivity of WS_2_-based sensors to CO even at high relative ambient humidity. Finally, we note that, similar to MoS_2_, also WS_2_-based sensors have been heavily studied using first principles calculations, specifically to study the influence of different atomic metal doping on improving the sensitivity and selectivity towards desired gases [309,310,311,312,313]. However, these studies will require confirmation with experiments and much work is still required to enable a stable fabrication of these films.

### 5.3. Phosphorene

Phosphorene refers to the ML of phosphorus and several allotropes of phosphorene have been investigated recently in their potential capacity for gas sensing. The most prominent one, which has also been investigated for the longest time due to the fact that it was synthesized the earliest, is black phosphorene. In fact, “phosphorene” usually is assumed to refer to “black phosphorene”. However, recently, blue and green phosphorene have also drawn attention in their potential use towards gas sensing applications. While there are claims being made with regards to fabricated blue phosphorene, most studies surrounding both blue and green allotropes are theoretical and rely on first-principles calculations. The differences between the various allotropes are given in Figure 26 from [314].

#### 5.3.1. Black Phosphorene

A ML of black phosphorus is commonly referred to as black phosphorene, or even simply phosphorene. The film possesses a honeycomb structure, while demonstrating p-type conductivity with a high carrier mobility and a band gap somewhere in the range of 0.3 eV and 1.9 eV, depending on the process used for its synthesis [315,316]. Much like several TMDs, black phosphorene has already been employed in the fabrication and testing of FETs [317,318] as well as for gas sensing applications [319]. It has been demonstrated using *ab initio* density functional theory (DFT) calculations that molecules such as CO, H_2_, H_2_O, and NH_3_ behave as electron donors, while NO, NO_2_, and O_2_ operate as electron acceptors, when adsorbed on the phosphorene surface [320]. While *ab initio* techniques have shown some propensity of H_2_ and CH_4_ adsorption on phosphorene [321], NO_2_ appears to exhibit the strongest interactions with the surface of the phosphorene film, highlighting its promise for future gas detector applications [220]. A black-phosphorus-based FET, according to experimental observations in [319], is capable of detecting levels of NO_2_ as low as 5 ppm in an argon environment, but the device’s response time is quite slow, i.e., on the scale of several minutes. The recovery time was shown to be even longer, taking over 30 min.

**Figure 27:**
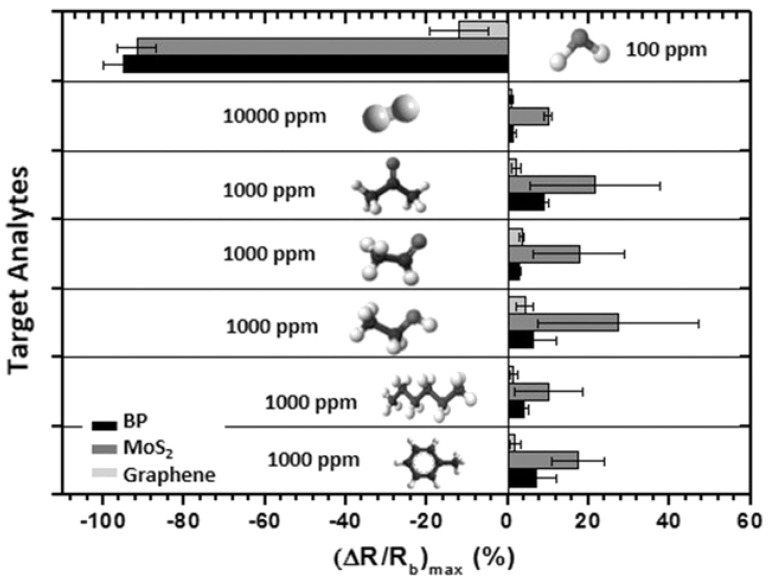
Change in the resistance of several 2D materials (phosphorene, MoS_2_, and rGO) when exposed to various ambient environments, showing an increased selectivity of phosphorene and MoS_2_ towards NO_2_. (Reprinted with permission from Cho et al. [322]).

A recent room-temperature study on the difference in response of various 2D films to the presence of NO_2_ was published by Cho et al. [322]. After looking into phosphorene, rGO, and MoS_2_, the authors found that phosphorene had the most significant response. Of particular interest was that phosphorene showed increased selectivity to NO_2_, even when compared to that of MoS_2_, as depicted in Figure 27.

**Figure 28:**
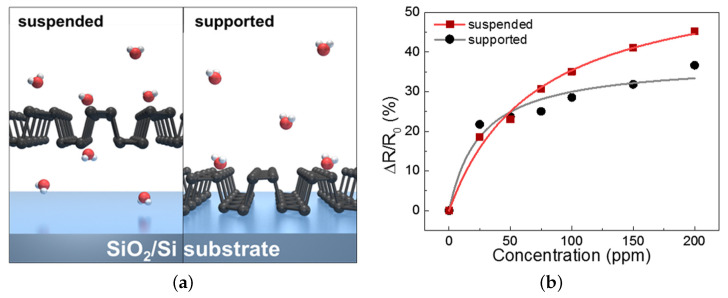
(**a**) Schematic of the CO gas sensing mechanism in the suspended and supported black phosphorene configurations. (**b**) The measured sensitivity in terms of μDR/R_N_2__ = |R_gas_− R_N_2__|/R_N_2__ at each gas concentration and their Langmuir isotherm fitting curves. (Reprinted with permission from Lee et al. [323]).

Another study looked at the sensing performance of suspended phosphorene films [323], noting that the suspended films demonstrated a faster desorption rate and a higher sensitivity to CO at concentrations above 100 ppm. When exposed to CO concentrations at 50 ppm or below, the suspended film performed the same as the one supported on a SiO_2_-on-silicon substrate. The results of this study are shown in Figure 28.

**Figure 29:**
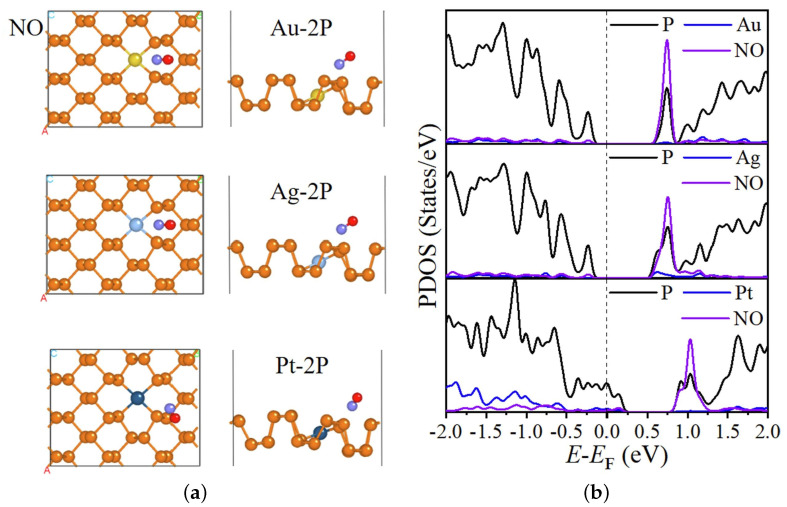
(**a**) Atomic configurations of Au-, Ag-, and Pt-doped phosphorene (with two P atoms substituted for one metal atom) with an NO gas molecule adsorbed. Corresponding PDOS are shown in (**b**). Balls in purple and red represent N and O atoms, respectively. The dashed line in the PDOS illustrates the Fermi level. (Reprinted with permission from Yang et al. [325]).

Similar to other chemiresistive gas sensors, studies on phosphorene are mainly concentrated on improving the selectivity using doping, primarily with noble metals. A recent study on Pt-decorated phosphorene has shown its propensity in applications as a room-temperature VOC sensor [324]. The authors in [324] show that in both armchair and zigzag directions, the Pt-decorated phosphorene outperforms the sensitivity of pristine phosphorene sheets for the detection of several VOCs, but the most significant difference is observed in the detection of ethanol and methanol. Yang et al. [325] recently also looked at the impact of metal doping on phosphorene for gas sensing using first-principles techniques. They observed that substituting two phosphorus atoms with gold, silver, or platinum is more stable than when only one phosphorus is substituted. They further propose the use of Au and Ag doping of phosphorene for the detection of NO gas [325]. In Figure 29a the most stable configuration of NO adsorption on phosphorene is shown to be above the center of the six-member ring, which includes the doping metal atom with the O atom away pointing away from the surface, for Au- and Ag-doped phosphorene. In the case of platinum-adsorbed phosphorene, the NO prefers to be adsorbed above the P–P bond and shows the strongest adsorption for the cases studied. All of these dopings appear to turn phosphorene into a metal, while introducing strain can turn them back into semiconductors, except in the case of Pt-doping.

**Figure 30:**
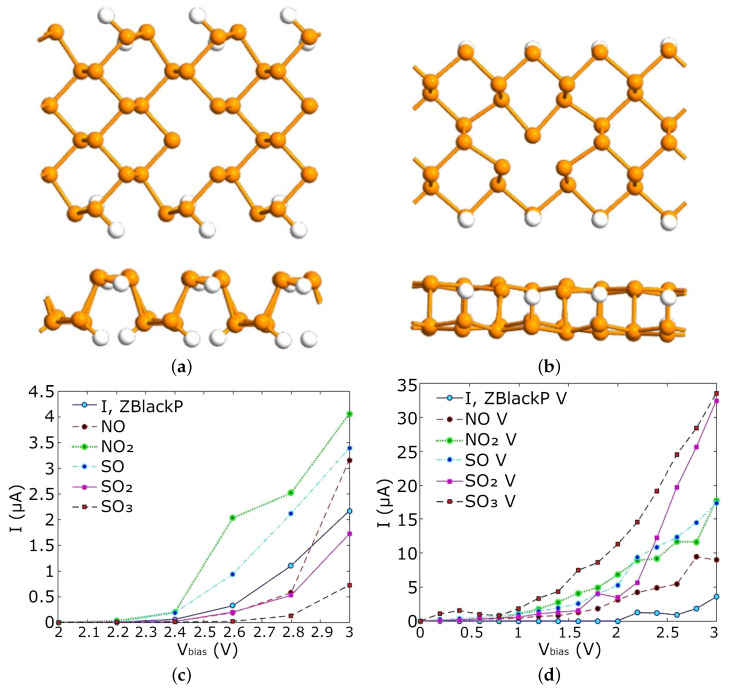
The optimized structures of vacancy defected black phosphorene MLs in their (**a**) armchair and (**b**) zigzag directions. The above row represents the top views and below row shows the side views of defected structures. The white and orange balls demonstrate H and P atoms, respectively. The current-voltage (IV) response for (**c**) pristine and (**d**) vacancy-defected phosphorene in the zigzag direction when exposed to various ambient gases. (Reprinted (adapted) with permission from Meshginqalam et al. [327]).

Another recent study by Zuluaga-Hernandez et al. analyzed the impact of decorating the phosphorene surface with oxygen atoms [326]. The authors studied the impact of interstitial and on-top oxygen on the electrical adsorption energies and the phosphorene’s response to the subsequent physisorption of CO_2_, NO, SO_2_, NH_3_, and H_2_S molecules. Their first-principles analysis suggests that the introduction of oxygen makes the film more suitable for the adsorption of these harmful molecules, while SO_2_ is shown to have the greatest difference in the electronic charge transfer. In [327], the authors analyzed the impact of vacancies in phosphorene nanoribbons on the detection of NO_2_ and SO_2_ molecules. The authors performed first-principles studies on vacancy-defected black and blue phosphorene. The results, provided in Figure 30, show that for all the tested molecules (NO, NO_2_, SO, SO_2_, and SO_3_), the phosphorene MLs with an induced vacancy (Zigzag BlackP V) outperformed the pristine phoshporene in their sensitivity. The authors also show that the zigzag direction outperforms the armchair direction in its sensitivity in all tested cases, except for SO adsorption.

#### 5.3.2. Blue Phosphorene

In the last few years, interest in blue phosphorene has significantly increased, most likely due to claims of its successful synthesis. Blue phosphorene is an allotrope of black phosphorus, which is formed by a single layer of phosphorus atoms more flatly arranged than in the case of black phosphorene [328]. A monolayer of blue phosphorene was theoretically shown to exhibit a carrier mobility of over 1000 cm^2^V^−1^s^−1^ [329] (the mobility of MoS_2_ is about 200 cm^2^V^−1^s^−1^, for comparison). Blue phosphorene also exhibits a fundamental indirect band gap of about 2 eV, which can be modified to a direct band gap through doping [330]. Blue phosphorene has been studied as a medium for gas sensing using first principles calculations in several studies [330,331,332]. First principles studies suggest that blue phosphorene can be an effective gas sensor using its pristine surface [333] in addition to an increased adsorption and sensitivity potential of phosphorus vacancy sites [327]. Further studies suggest that specific doping can improve sensitivity and selectivity towards desired molecules [330,332]. Recent research suggest that covalent bonds do not form between small gas molecules (e.g., H_2_O, SO_2_, NH_3_, H_2_S, O_2_) on pristine blue phosphorene and that physical adsorption takes place. The authors in [333] suggest that the physical adsorption of gas molecules can be used to tune the band gap of blue phosphorene and its work function. However, strong adsorption and significant changes occur as a result of adsorption on vacancy defects, which was recently described for NO_x_ and SO_x_ sensing [327].

Furthermore, numerous recent studies suggest that metal- and non-metal-doped blue phosphorene exhibits the potential for highly sensitive and selective gas sensing [334,335,336]. One particular study even looked at the impact of both vacancies and aluminum doping on blue phosphorene’s response to interactions with an ambient molecule [337]. The authors therein observe that acetylene, O_3_, SO_3_, H_2_Se, and SCl_2_ show enhanced sensing properties, when the film is doped or in the presence of a vacancy. In fact, many of these molecules appear to adsorb chemically under these conditions: Acetylene, H_2_Se, and SCl_2_ chemisorb on a doped blue phosphorene surface, while acetylene, O_3_, and SO_3_ chemisorb on the single P vacancy.

The positive first-principles results obtained for blue phosphorene appear to show some promise in its eventual application for gas sensing devices. Several years ago, the promise of blue phosphorene devices was very high as the material appeared to be synthesized, resulting in high hopes for its potential use in gas sensing, bio-sensing, and a host of other applications [338,339,340,341]. However, several recent studies have cast doubt over the experimental results achieved with this material, especially relating to highly sensitive gas sensing [342]. While experimental realization of 2D blue phosphorene grown on Au(111) was reported previously, the origin of the experimentally observed superlattice was actually revealed to be a two-dimensional porous gold–phosphorus network with blue phosphorene sub-units linked by gold atoms [343,344]. Some groups are currently attempting to perform silicon intercalation on blue phosphorene–gold alloys to overcome this problem and obtain pristine ML blue phosphorene [343]. However, currently, there does not exist any means to synthesize stable blue phosphorene MLs.

### 5.4. MXenes

MXenes are a class of 2D inorganic compounds with a narrow band gap on the order of about 0.2 eV [345]. The material consists of a few-atoms-thick layer of transition metal carbides, nitrides, or carbonitrides. The first such film, namely, 2D Ti_3_C_2_, was synthesized in 2011 by Naguib et al. [346] by exfoliating Ti_3_AlC_2_. The exfoliation process includes replacing Al atoms with OH after immersion in HF and subsequent reactions. The hydrogen bonds are then broken, and the nanosheet is separated after sonication in methanol.

MXenes have merged onto the ever-increasing scene of 2D materials relatively recently, especially when compared to graphene and TMDs. However, this younger member of the 2D materials family has attracted significant attention for its physical, chemical, and electronic properties, which may be suitable for many relevant applications [347]. When considering MXene films for gas sensing, investigations have shown that they enhance chemical functionality for gas adsorption while enjoying the benefits of large surface-to-volume ratio, as noted with other 2D materials [348].

Until recently, only a handful of results have been published which include an experimental display of the gas sensing potential of MXenes. The first theoretical studies showing the potential to use 2D MXenes for gas sensing were published in 2019 and dealt with the adsorption of NO and CO on pristine MXene surfaces by Yang et al. [349]. These theoretical predictions lead to experimental investigations on using alkalized V_2_CT_x_ for NO sensing [350]. Further studies supported the promise of using MXene films for the detection of various gases, including VOCs [351,352], H_2_ [352], CO_2_ [353], NO_2_ [354,355], and H_2_S [356]. The CO_2_ detection using a Mo_2_CT_x_-based sensor on different substrates, i.e., glass, crystalline silicon (cSi) and porous silicon (pSi), is shown in Figure 31 from [353]. From the figure, we note that the glass and crystalline silicon substrates exhibit optimal sensitivities at about 200 °C, while porous silicon seems to not have this limitation and the sensing response increases as the temperature increases. This suggests that the interface between the substrate and the MXene film should be further studied, as it may impact the sensing mechanism.

**Figure 32:**
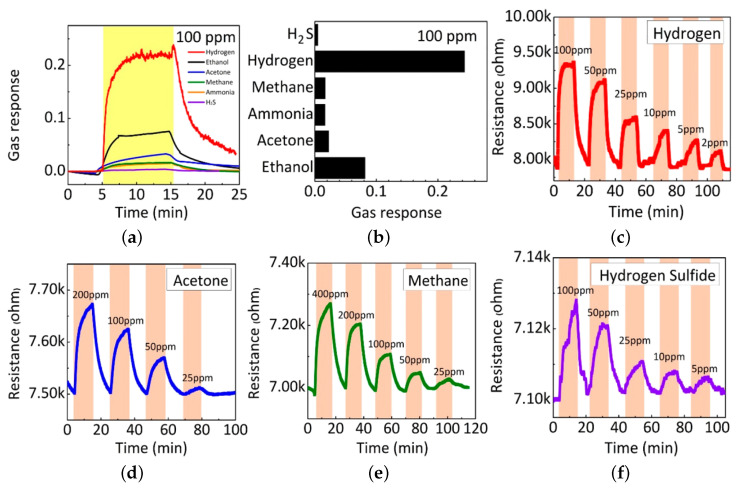
Gas sensing properties of a V_2_CT_x_ sensor at room temperature. (**a**) The compiled resistance variation and (**b**) gas response toward 100 ppm hydrogen, ethanol, acetone, methane, ammonia, and H_2_S. Real-time sensing response of V_2_CT_x_ gas sensors at varying concentrations of (**c**) hydrogen, (**d**) acetone, (**e**) methane, and (**f**) H_2_S. (Reprinted with permission from Lee et al. [352]).

Several authors have also recently shown that modifying the MXene surface, whether through metal functionalization, by combining with SMO nanostructures, or by other means, can improve the sensitivity of these materials. Zhao et al. [354] managed to improve the NO_2_ sensing performance of the MXene Ti_3_C_2_T_x_ by μg-Poly(l-glutamic acid) modification. Through this modification, the response increased by more than 10-fold compared to non-modified Ti_3_C_2_T_x_ films. Fan et al. [355] was able to develop fast and recoverable NO_2_ sensors through the assembly of ZnO on MXene Ti_3_C_2_T_x_ under UV illumination. Wang et al. [357] developed a self-powered NO_2_ sensor using a Ti_3_C_2_T_3_/WO_3_ film, showing an excellent sensitivity of over 500% for 50 ppm NO_2_. Many other studies followed, which further confirm the improved sensitivity towards NO_2_ with hybrid MXene/SMO films, mainly using SMOs WS_3_ and ZnO [358,359,360,361]. Xu et al. [356] explored H_2_S sensing using Ag-functionalized Ti_3_C_2_T_x_. The functionalization appears to improve the sensitivity over those reported on a pristine MXene film, albeit using vanadium carbine MXene [352]. A summary of a VOC gas sensor achieved with V_2_CT_x_ MXene at room temperature is provided in Figure 32 [352]. While the film responds the most to hydrogen, it is clear that it is able to detect several VOCs with sufficient sensitivity. However, ensuring a selective response is still out of reach.

**Figure 33:**
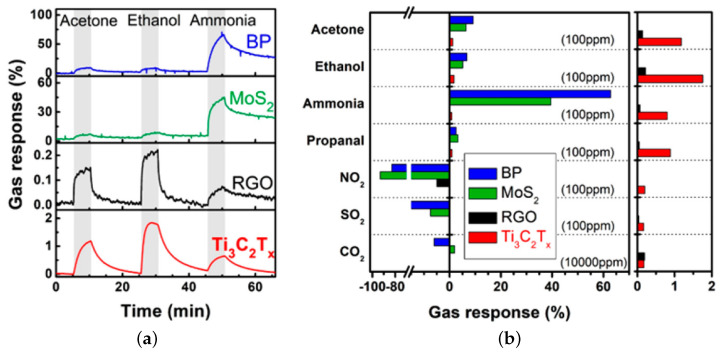
Measured gas response of Ti_3_C_2_T_x_ sensors compared with sensors based on other 2D materials, such as black phosphorene (BP), MoS_2_, and rGO. (**a**) Real-time gas response behavior of these sensors to 100 ppm of target gases. (**b**) Maximum resistance change after exposure to 10 ppm acetone, ethanol, ammonia, propanal, NO_2_, SO_2_, and 10,000 ppm CO_2_. Inset to the right displays a magnified scale to be able to see Ti_3_C_2_T_x_ and rGO. (Reprinted (adapted) with permission from Kim et al. [362]; further permissions related to this material excerpted should be directed to the ACS).

Kim et al. [362] compared the performance of the MXene Ti_3_C_2_T_x_ to those of rGO, MoS_2_, and black phosphorene (BP) for the detection of acetone, ethanol, ammonia, propanal, NO_2_, SO_2_, and CO_2_, with results shown in Figure 33. From these results, we can conclude that, overall, blue phosphorene and MoS_2_ seem to offer the highest sensitivities to a broad range of gases. However, while black phosphorene and MoS_2_ have the highest sensitivity towards ammonia detection, the signals are not very selective against NO_2_. The MXene Ti_3_C_2_T_x_, on the other hand, has a lower total sensitivity towards ammonia, but it is highly selective against NO_2_.

### 5.5. Two-Dimensional Heterojunctions

This review is mainly concerned with single-crystal 2D semiconductor films for gas sensing technologies with a high potential for CMOS integration. However, there has been significant interest recently in the application of 2D heterostructures in order to increase the sensitivity or selectivity of these thin layers [363,364]. The use of heterostructures is not new and has also been applied to SMO-based chemiresistive sensors in the past [365,366]. Nevertheless, some recent achievements in 2D heterostructure gas sensors are presented here.

#### 5.5.1. Fabrication of 2D Heterostructures

It should be noted that any stacking of multiple 2D materials introduces further fabrication complexity and reduces the probability of time- and cost-efficient CMOS integration. However, there have been reports of controllable fabrication of 2D heterostructures using mechanical stacking or direct-growth methods [364]. While mechanical stacking is not a process which can easily be incorporated in a CMOS foundry, direct-growth methods using CVD have better potential here [367,368,369]. To date, a wide variety of 2D heterostructures have been fabricated using CVD with a wide ranging set of properties which are shown to depend on specific parameters of the process itself, such as the type of species, the precursor dosage, the type and flow rate of the carrier gas, the temperature, and the duration of the chemical reaction. Nevertheless, a comprehensive understanding of the fabrication methods and a link between process parameters and the growth variables of heterostructures has not been made, and finding these remains a great challenge. Furthermore, just like in the CVD deposition of single 2D films, heterostructure deposition requires high temperatures between 650 °C and 850 °C [367]. In the meantime, MBE [370,371] and ALD [192] have also been successfully applied for the growth of 2D semiconductor heterostructures.

#### 5.5.2. Gas Sensing with 2D Heterostructures

**Figure 34:**
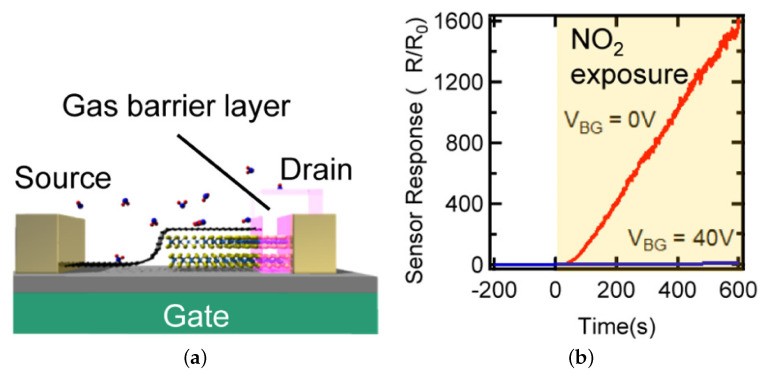
(**a**) Gas sensing FET based on a vdW heterojunction consisting of graphene and MoS_2_. (**b**) Sensor response to a 1 ppm exposure to NO_2_ when operating in reverse-bias conditions. (Reprinted with permission from Tabata et al. [374]).

The first successful application of 2D heterostructures in gas sensing took place quite recently in 2015, by Cho et al. [372]. The presented graphene–MoS_2_ heterostructure, synthesized using CVD (for graphene) and mechanical exfoliation (for MoS_2_), demonstrated a gas sensor with a sensitivity towards NO_2_ and NH_3_ at 1.2 ppm and 5 ppm, respectively, when operating at 150 °C. Noble metal nanoparticles were also used in such nanostructures to tailor the sensing response. The use of palladium, for example, was shown to increase the sensitivity of response towards NH_3_ while reducing the response to NO_2_; aluminum had the reverse effect, whereby its use reduced the sensitivity towards NH_3_ and increased the response to NO_2_ [373]. The highest sensitivity which was achieved with 2D heterostructure gas sensors was presented by Tabata et al. [374] using a graphene–MoS_2_ device, shown in Figure 34a. The authors demonstrated a gas sensor which is able to detect NO_2_ down to 1 ppm with a change in resistance by a factor greater than 10^3^, shown in Figure 34b. The device is based on a 2D heterojunction FET with a back-gate and a Schottky gas barrier layer.

#### 5.5.3. Illuminated 2D Heterojunction Gas Sensors

**Figure 35:**
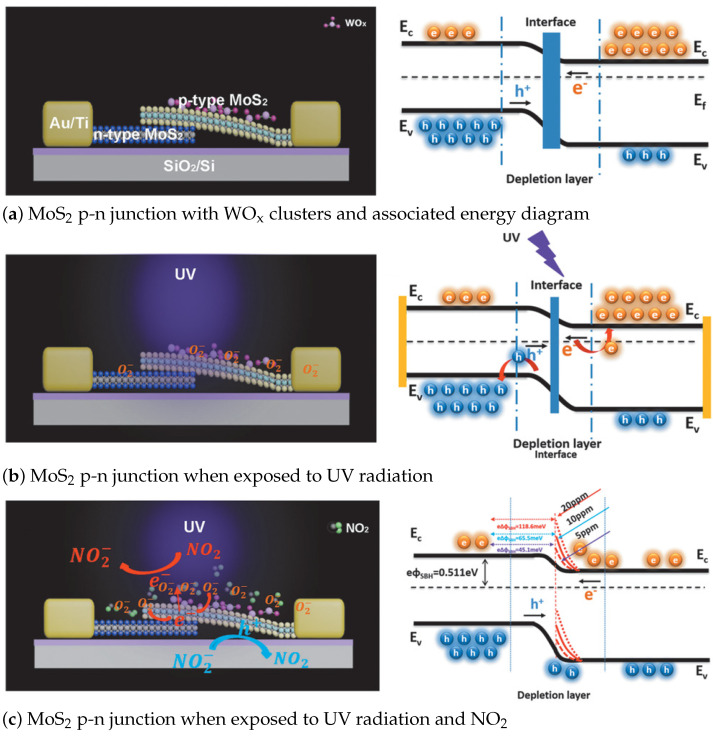
Schematic structures and band diagrams of a WO_x_-decorated MoS_2_ heterojunction (**a**) before and (**b**) after UV exposure and (**c**) NO_2_ exposure. (Reprinted with permission from Zheng et al. [375]).

Several researchers have taken to combining many of the concepts discussed in this review in order to fabricate highly sensitive and selective sensors. Zheng et al. [375] have used an n–p MoS_2_ heterostructure, decorated with WO_x_ particles and added UV illumination to show a promising NO_2_-selective gas sensor. A schematic and energy band diagram of this MoS_2_ p-n heterostructure based sensor under UV irradiation is shown in Figure 35, from [375]. The sensor operates in a similar way as the SMO sensor. First, oxygen species are adsorbed onto the surface. The subsequent reaction of the adsorbed oxygen with ambient NO_2_ gas removes some of these surface oxygen ions. However, the impact of the changing surface charges is not the reduction of a simple surface depletion layer. Rather, the process is more complex. When n-type and p-type MoS_2_ are contacted, electrons will transfer from the n- to the p-type while holes transfer from the p- to the n-type MoS_2_, cf. Figure 35a. This makes the p-type (n-type) MoS_2_ more positive (negative) and forms a depletion layer at the interface. When this p–n junction and depletion layer is exposed to UV radiation, a large number of photogenerated free carriers are produced, which then move towards the n-type (electrons) and p-type (holes) MoS_2_, cf. Figure 35b. This process provides more charge carriers to the surface, which can participate in the gas sensing process by exchanging charges with adsorbed oxygen ions. The introduction of NO_2_ gas molecules under UV radiation causes some of the oxygen ions to be removed, increasing the Schottky barrier height at the surface, cf. Figure 35c.

### 5.6. Summary

In Table 3, we summarize the recent (within the previous 5 years) progress in applying 2D semiconductors and 2D heterojunctions towards the sensing of the four most relevant air pollutants, as noted by the WHO and summarized in Table 1. From this summary, we can observe that the sensing is primarily carried out at room temperature (RT) or temperatures far below those of SMO-based gas sensors. However, from the major pollutants, it appears that NO_2_ is the one which is the most studied and which has the highest sensitivities in 2D-material-based gas sensors. It appears that most sensors, which show a high sensitivity towards CO, are often either decorated with noble metals or are combined with another material, often an SMO. Only black phosphorene appears to show an increased sensitivity towards CO, although it is not as sensitive as many SMO studies and not as selective as optical NDIR alternatives. The sensors based on SO_2_ all appear to include some form of heterojunction between SMO films and a 2D layer, meaning that 2D semiconductors alone may not provide a sufficiently strong response. The similar is the case with ozone (O_3_) where several studies combine rGO with ZnO for heightened sensing.

Furthermore, as we noted in a previous section, there are discrepancies in how the sensitivities are being reported in literature, whereby some authors choose to compare the change in the electrical properties when a target gas is introduced in the air ambient environment, while others use an inert N_2_ environment. Therefore, these sensors cannot be compared directly and more information is required before any conclusions can be made about their potential applicability in real-world conditions.

We should note that we included only experimentally observed gas sensing responses in Table 3 and ignored all theoretical studies, including *ab initio* calculations, from which there are plenty. The table gives a relatively bleak perspective on the use of pristine 2D materials for the detection of CO, SO_2_, or O_3_, meaning that SMO and optical solutions for these are likely to persist for the time being. However, the discussion from the previous sections gives hope in potentially replacing SMO sensors for the detection of VOCs, as well as methanol, ethanol, ammonia, and NO_2_.

## 6. Conclusions

In this review, we examine the development and current state of research of semiconductor-based gas sensors, focusing in particular on potentially CMOS integration-friendly technologies such as 2D-based materials. The SMO-based gas sensor, considered the current workhorse of semiconductor-based chemiresistive gas sensor technologies, requires high temperatures to initiate the surface reactions which result in the sensing response, making it difficult to fabricate and prone to high mechanical instability. Therefore, alternatives at lower temperatures are desired, where 2D materials seem to hold the most promise. Even at ambient temperature, their sensitivity is extraordinarily large due to their extremely high surface-to-volume ratio. However, some ongoing issues still need to be resolved before gas sensors based on 2D materials can be widely used and commercialized. The alternative room temperature solutions involve optical signals, either by designing an NDIR sensor based on the Beer-Lambert law or by introducing an additional UV illumination to SMO sensors. In both cases, CMOS integration is not feasible, which is why continued interest in 2D-material-based gas sensors persists.

Due to graphene’s lack of a band gap, research attention has shifted towards alternative 2D materials, including those based on graphene, such as GO and rGO, as well as other materials, such as TMDs, phosphorene, and the MXene family of materials. The closest to production-ready devices are those based on GO and rGO, although much more work needs to be performed to enable the mass production of devices based on these materials. Specifically, we need to make sure that film synthesis can be performed cheaply, efficiently, and with a high reproducibility of predictable device behavior. Especially reduced graphene oxide fabrication can be very imprecise as it requires a multi-step process of graphene oxidation, followed by its reduction. For TMDs, due to considerable advancements achieved in the synthesis of these materials via CVD in recent years, they are currently considered to represent the future of digital transistors and sensing. A particular concern with TMDs materials is that, in their pristine form, they exhibit a low carrier mobility which is only increased through the introduction of vacancies, dopants, and point defects. However, introducing defects comes with a plethora of other concerns, such as poor reliability and operational drift. An additional concern is the effects of undesirable interaction between the potentially defected TMD surface and ambient molecules, such as humidity. Additionally, it is not yet fully understood, how the conductivity of these films is affected by defects and vacancies, meaning that continued research and investigations are necessary. Phosphorene research is still in its relative infancy and is primarily rooted in DFT and other first-principles calculations. Nevertheless, phosphorene shows particular promise for future applications towards the selective sensing of gas molecules containing nitrogen.

When looking into the fabrication of relevant 2D materials, it is clear that many options are being studied, including CVD, PVD, MBE, and ALD. From these options, CVD and MBE can produce large crystalline flakes, but they require high temperatures and equipment which is not readily available in a standard CMOS foundry. The other processes, PVD sputtering and ALD, can be performed at low temperatures, but they produce highly polycrystalline films with poor stoichiometry. These can be repaired with a high-temperature annealing step, thereby negating their initial benefits for CMOS integration.

We note that 2D films have many advantages for gas sensing, when compared to bulk materials; however, further studies and increased cooperation between theoretical and experimental scientists are essential to fully realize and capitalize on these advantages. The fabrication of predictable black phosphorene layers has become relatively straight-forward, albeit expensive and not compatible toCMOS fabrication. However, blue and green phosphorene are in their infancy, and whether these films will ever be successfully synthesized is still up for debate. The past few years have seen enormous efforts and progress in the synthesis of various MXene films, and their potential for gas sensor applications has already become quite clear. However, MXenes have a relatively narrow bandgap, when compared to phosphorene or the TMD alternatives, meaning that making digital logic transistors or FET-based sensors with these materials might be challenging.

The modification of 2D materials with various composites, dopants, vacancies, hybrids, and heterostructures is shown to be the main means to improve the gas sensing performance, such as sensitivity and selectivity, even at room temperature. However, the added complexity required to synthesize or engineer composite or functionalized films may be too expensive or cumbersome to provide a realistic means towards mass production and integration in CMOS technology. The progress until now has been breathtaking, but much still needs to be done if 2D-based devices are to dethrone the SMO gas sensor or if they will even come close to taking a bite out of silicon’s dominance in the digital logic landscape.

## Figures and Tables

**Figure 14:**
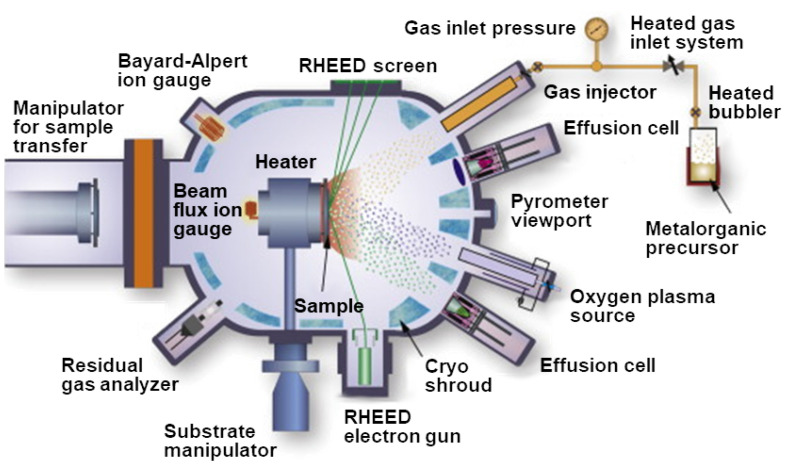
Schematic of the MBE setup for growing complex oxides. Metals evaporating from effusion cells are oxidized, exposing them to ozone, molecular oxygen, or atomic oxygen generated by an RF plasma source. (Reprinted (adapted) with permission from Engel-Herbert [186]).

**Figure 19:**
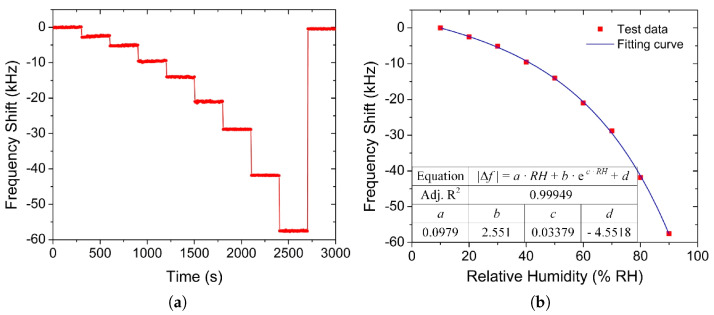
Detection of humidity with a piezoelectric micromachined ultrasonic transducer with a GO layer is shown. (**a**) The shift in frequency as a result of varying relative ambient humidity from 10% to 90% is shown. (**b**) The results of the relative humidity response from (**a**) fit nicely to an exponential equation. (Reprinted with permission from Sun et al. [226], CC-BY 4.0).

**Figure 26:**
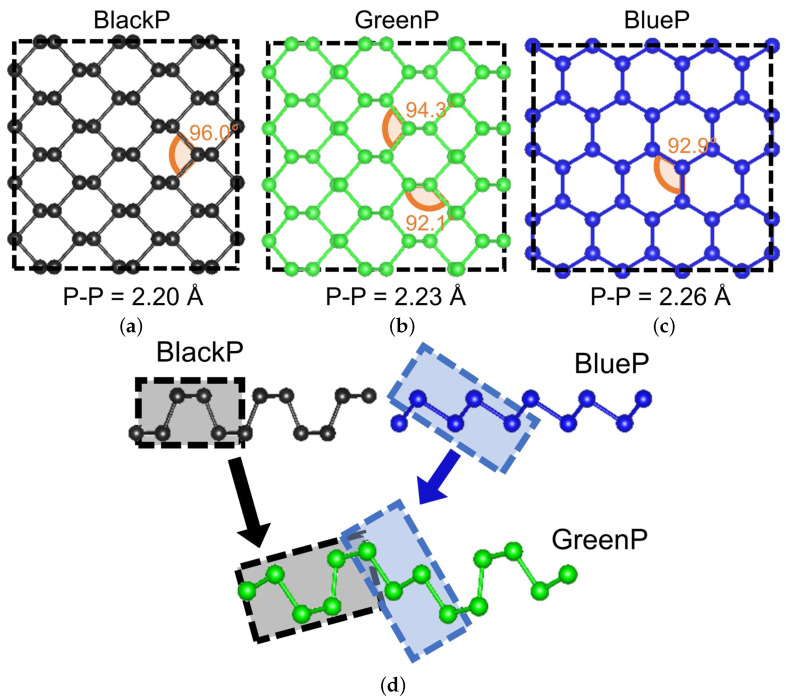
The relaxed atomic structures of different polymorphs of phosphorene, including (**a**) Black, (**b**) Green, and (**c**) Blue. (**d**) The magnification of atomic structure of Black and Blue Phosphorene, indicating that Green Phosphorene is derived from the mixture of the black and the blue phases. (Reprinted (adapted) with permission from Kaewmaraya et al. [314]).

**Figure 31:**
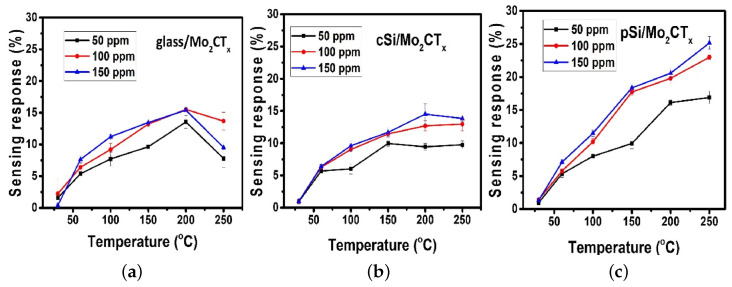
Working temperature versus gas sensing response for (**a**) glass/Mo_2_CT_x_, (**b**) cSi/Mo_2_CT_x_, and (**c**) pSi/Mo_2_CT_x_ sensors for different CO_2_ concentrations. (Reprinted with permission from Thomas et al. [353]).

**Table 1:** Different international standards for hazardous levels of exposure to some common pollutants in terms of minutes (min), hours (h), months (m), or years (yr). For reference, 1 ppm is equivalent to 1000 µg/m^3^ or 1 mg/m^3^ in volume.

	WHO		EPA		EC		MEP	
CO	100 mg/m^3^ 15 mg/m^3^ 10 mg/m^3^ 7 mg/m^3^	(15 min) (1 h) (8 h) (24 h)	35 ppm 9 ppm	(1 h) (8 h)	10 mg/m^3^	(8 h)	10 mg/m^3^ 4 mg/m^3^	(1 h) (24 h)
NO_2_	200 µg/m^3^ 40 µg/m^3^	(1 h) (1 yr)	100 ppb 53 ppb	(1 h) (1 yr)	200 µg/m^3^ 40 µg/m^3^	(1 h) (1 yr)	200 µg/m^3^ 80 µg/m^3^ 40 µg/m^3^	(1 h) (24 h) (1 yr)
O_3_	100 µg/m^3^	(8 h)	75 ppb	(8 h)	120 µg/m^3^	(8 h)	200 µg/m^3^ 160 µg/m^3^	(1 h) (1 yr)
SO_2_	500 µg/m^3^ 20 µg/m^3^	(10 min) (24 h)	75 ppb 500 ppb	(1 h) (3 h)	350 µg/m^3^ 125 µg/m^3^	(1 h) (24 h)	500 µg/m^3^ 150 µg/m^3^ 60 µg/m^3^	(1 h) (24 h) (1 yr)
PM_2.5_	25 µg/m^3^ 10 µg/m^3^	(24 h) (1 yr)	35 µg/m^3^ 12 µg/m^3^	(24 h) (1 yr)	25 µg/m^3^	(1 yr)	75 µg/m^3^ 35 µg/m^3^	(24 h) (1 yr)
PM_10_	50 µg/m^3^ 20 µg/m^3^	(24 h) (1 yr)	150 µg/m^3^	(24 h)	50 µg/m^3^ 40 µg/m^3^	(24 h) (1 yr)	100 µg/m^3^ 50 µg/m^3^	(24 h) (1 yr)

**Table 2:** Ranked properties of currently investigated gas sensor technologies. 4 = Excellent, 3 = Good, 2 = Fair, 1 = Poor.

Parameter	SMO	CP	PE	EC	TP	PI	IR
Sensitivity	4	3	4	3	1	4	4
Accuracy	3	3	4	4	3	4	4
Selectivity	2	1	2	3	1	2	4
Speed	4	3	4	2	3	4	2
Stability	3	3	3	1	3	4	3
Durability	3	3	2	2	3	4	4
Power	4	4	2	3	3	1	2
Cost	4	4	3	3	3	2	2
Footprint	4	3	3	2	3	4	1

**Table 3:** Summary of 2D materials and heterostructures for gas sensing of the air pollutants noted as the most relevant by the WHO, cf. Table 1, published in the last few years. In the heading, Conc., Temp., Sens. def, Sens., and Ref. refer to the Concentration, Temperature, Sensitivity definition, Sensitivity, and Reference, respectively. In the table, I, R, and G refer to the current, resistance, and conductance, respectively; gas, air, and N_2_ refer to the target gas, air, and inert N_2_ ambient conditions, respectively.

Material	Gas	Conc. (ppm)	Temp.	Sens. Def.	Sens.	Ref.
WS_2_	CO	50	RT	R_gas_/R_air_	2	[308]
Ru-WS_2_	CO	50	RT	R_gas_/R_air_	3.7	[308]
TiO_2_/rGO	SO_2_	20	RT	|I_gas_-Iair|/I_air_	3.74%	[256]
porous rGO	NO_2_	2	100 °C	|G_gas_-G_N_2__|/G_N_2__	2400%	[250]
rGO/chitosan	NO_2_	1	RT	|I_gas_-Iair|/I_air_	23%	[236]
rGO/chitosan	NO_2_	10	RT	|I_gas_-Iair|/I_air_	67%	[236]
rGO/chitosan	NO_2_	100	RT	|I_gas_-Iair|/I_air_	113%	[236]
Mo_2_CT_x_ on polySi	CO_2_	50	250	|R_gas_-Rair|/R_air_	17%	[353]
alkalized V_2_CT_x_	NO_2_	5	RT	|R_gas_-Rair|/R_air_	5%	[350]
alkalized V_2_CT_x_	NO_2_	50	RT	|R_gas_-Rair|/R_air_	57%	[350]
polypyrrole-GO	CO	50	RT	|R_gas_-R_N_2__|/R_N_2__	8%	[251]
polypyrrole-GO	CO	300	RT	|R_gas_-R_N_2__|/R_N_2__	44%	[251]
SnO_2_/MoS_2_	SO_2_	10	100 °C	R_gas_/R_air_	10	[290]
rGO-ZnO	O_3_	0.1	300 °C	R_gas_/R_air_	49.6	[257]
MoS_2_/CNT	SO_2_	0.5	RT	|R_gas_-Rair|/R_air_	0.22%	[376]
MoS_2_/CNT	SO_2_	3	RT	|R_gas_-Rair|/R_air_	1.8%	[376]
phosphorene	NO_2_	0.1	RT	|I_gas_-I_N_2__|/I_N_2__	88%	[377]
UV p/n-MoS_2_	NO_2_	20	RT	R_gas_/R_air_	0.95	[375]
Au-WS_2_	CO	1	RT	R_gas_/R_air_	1.2	[305]
Au-WS_2_	CO	10	RT	R_gas_/R_air_	1.23	[305]
Au-WS_2_	CO	50	RT	R_gas_/R_air_	1.4	[305]
MoS_2_	CO	100	200 °C	|R_gas_-Rair|/R_air_	28%	[378]
MoS_2_/Graphene	NO_2_	10	200 °C	|R_gas_-Rair|/R_air_	69%	[379]
SnS	NO_2_	3.75	60 °C	|R_gas_-Rair|/R_air_	68%	[380]
Au on 2L-MoS_2_	NO_2_	10	RT	|I_gas_-I_N_2__|/I_N_2__	60%	[381]
WS_2_	CO	50	RT	R_air_/R_gas_	1.05	[264]
Au on WS_2_	CO	50	RT	R_air_/R_gas_	1.48	[264]
WS_2_	NO_2_	5	RT	|R_gas_-Rair|/R_air_	68.4%	[382]
UV-MoTe_2_	NO_2_	0.02	RT	|G_gas_-G_N_2__|/G_N_2__	18%	[383]
WSe_2_	NO_2_	10	RT	|I_gas_-I_N_2__|/I_N_2__	162%	[384]
rGO/graphene	NO_2_	10	RT	|R_gas_-Rair|/R_air_	15%	[245]
Graphene/MoS_2_	NO_2_	1	RT	|R_gas_-R_N_2__|/R_N_2__	>10^3^	[374]
Ti_3_C_2_T_x_	NO_2_	100	RT	|R_gas_-R_N_2__|/R_N_2__	0.2%	[362]
Ti_3_C_2_T_x_	SO_2_	100	RT	|R_gas_-R_N_2__|/R_N_2__	0.17%	[362]
Ti_3_C_2_T_x_	CO_2_	10,000	RT	|R_gas_-R_N_2__|/R_N_2__	0.13%	[362]
rGO/ZnO	O_3_	0.7	RT	|R_gas_-Rair|/R_air_	99%	[385]
phosphorene	CO	25	RT	|R_gas_-R_N_2__|/R_N_2__	22%	[323]
phosphorene	CO	200	RT	|R_gas_-R_N_2__|/R_N_2__	37%	[323]

## Abbreviations

2D	two-dimensional
3D	three-dimensional
ADC	analog-to-digital converter
ALD	atomic layer deposition
BEOL	back-end-of-line
BioFET	biologically sensitive field-effect transistor
BP	black phosphorene
CCFET	capacitively-coupled field-effect transistor
CMOS	complementary metal oxide semiconductor
CMP	chemical mechanical polishing
CNT	carbon nanotube
CoT	Cloud of Things
CVD	chemical vapor deposition
DFT	density functional theory
EC	European Commission
EPA	Environmental Protection Agency
FEOL	front-end-of-line
FET	field-effect transistor
GO	graphene oxide
hBN	hexagonal Boron Nitride
HFGFET	horizontal floating-gate field-effect transistor
I/O	input/output
InSe	indium selenide
IoE	Internet of Everything
IoT	Internet of Things
IR	infrared
ISFET	ion-sensitive field-effect transistor
IV	current-voltage
LED	light-emitting diode
MBE	molecular-beam epitaxy
MEMS	microelectromechanical system
MEP	Ministry of Environmental Protection
MGFET	metal gate field-effect transistor
ML	monolayer
MOCVD	metal-organic chemical vapor deposition
MOSFET	metal-oxide semiconductor field-effect transistor
NDIR	nondispersive infrared
PDOS	partial density of states
PEALD	plasma-enhanced atomic layer deposition
PECVD	plasma-enhanced chemical vapor deposition
ppb	parts per billion
ppm	parts per million
PVD	physical vapor deposition
RAM	random access memory
RF	radio-frequency
RGA	residual gas analyzer
rGO	reduced graphene oxide
RH	relative humidity
RHEED	reflection high-energy electron diffraction
ROM	read-only memory
RT	room temperature
SGFET	suspended gate field-effect transistor
SMO	semiconductor metal oxide
SoC	system-on-chip
SOI	silicon-on-insulator
TFT	thin-film transistor
TMD	transition metal dichalcogenide
US	United States
UV	ultraviolet
VCSEL	vertical-cavity surface-emitting laser
vdW	van der Waals
VOC	volatile organic compound
WHO	World Health Organization
